# Key Dietary Flavonoids: Insights into Their Structural Characteristics, Antioxidant and Anti-Inflammatory Activities, and Potential as Neuroprotective Agents

**DOI:** 10.3390/molecules31010154

**Published:** 2026-01-01

**Authors:** Mirtha Navarro-Hoyos, Rajesh Bisoyi, Puja KC, Nicholas Lutz, Mary Ruxsarash

**Affiliations:** 1 Chemistry Department, Georgetown University, Washington, DC 20057, USA; rkb60@georgetown.edu (R.B.); pk787@georgetown.edu (P.K.); nsl51@georgetown.edu (N.L.); mkr84@georgetown.edu (M.R.); 2 Bioactivity & Sustainable Development (BIODESS) Group, Department of Chemistry, University of Costa Rica (UCR), San Jose 2060, Costa Rica

**Keywords:** flavonoids, flavonols, flavones, flavan-3-ols, proanthocyanidins, anthocyanins, antioxidant activity, anti-inflammatory effects, neuroprotection

## Abstract

Among natural products, polyphenols have drawn special attention due to their antioxidant properties and inherent anti-inflammatory mechanisms. Within polyphenols, flavonoids are particularly important because of their availability in natural sources and promising research results in both in vitro and in vivo studies. The wide range of potential health benefits associated with these molecules has led to an increase in consumption, both as ingredients and extracts, in dietary supplements. Four types of flavonoids that have experienced significant attention are flavonols, flavones, anthocyanins, and flavan-3-ols, including proanthocyanidins. The increasing consumer awareness of the cognitive health benefits associated with the antioxidant and anti-inflammatory properties of these flavonoids has led to a rise in demand for these molecules in products that promote healthy aging. This review aims to provide an overview of these four types of flavonoids, focusing on their structure, antioxidant role, anti-inflammatory properties and potential neuroprotective effects, addressing key health priorities for consumers.

## 1. Introduction

Polyphenols have experienced a growing market demand among natural bioactives due to consumers’ increasing awareness of their potential health benefits as dietary supplement ingredients. Among polyphenols, flavonoids have particularly attracted the attention of consumers focused on healthy aging, prioritizing neuroprotective effects and cognitive health, which are linked to their antioxidant and anti-inflammatory properties. This prioritization has contributed to the sustained demand for these flavonoids, especially flavonols, flavones, anthocyanins, and flavan-3-ols, including proanthocyanidins in the past decade.

The global polyphenols market, as ingredients in dietary supplements, was estimated to be $2.14 billion in 2023 [1]. Sales data from the US between 2015 [2] and 2023 [3] indicates that cranberry products, rich in proanthocyanidins, reached $97.5 million in annual sales in 2023, while elderberry products, rich in anthocyanins, reached $201 million with Compound Annual Growth Rates (CAGR) of 3.5% and 26.9%, respectively. The Dietary Supplement Label Database (DSLD) of the National Institutes of Health (NIH) shows a steady increase in the number of registered products containing these types of flavonoids, with over twenty-five thousand botanicals currently listed [4].

For example, grape seed, cranberry and green tea supplements, which are all rich sources of proanthocyanidins, as well as flavonols like quercetin, hyperoside and rutin, can be found in over twelve thousand registered botanical products [4] with CAGR ranging between 19% and 21% in the last ten years. Additionally, products containing flavones such as apigenin and luteolin, passionflower products high in iso(vitexin) and (iso)orientin, and elderberry, black currant, bilberry, and blueberry products containing anthocyanins exhibit CAGRs exceeding 22% in the last decade.

Among the research on potential health benefits, the antioxidant effects of flavonoids have been attributed to their ability to inhibit radicals through various mechanisms, which is an important common characteristic. However, it has been found that the intrinsic structural differences among these flavonoids impact the degree of antioxidant activity and the type and extent of modulation in inflammatory processes, including factors such as cytokines and different signaling pathways. These properties in turn influence the type and level of potential neuroprotective effects related to cognitive improvement and neurodegenerative diseases [5,6].

Understanding these properties is crucial for the proper use and potential health applications of these flavonoids. Furthermore, the market is shifting towards extracts with multiple herbs and combinations of products, reflecting consumer preferences [3]. This highlights the importance of understanding the scientific knowledge behind the intake of these molecules to avoid potential interactions and ensure efficacy and safety.

Therefore, this review aims to provide an overview of these four types of flavonoids with a steady increase in consumption trend and high market growth, focusing on their structure, antioxidant activity, inflammatory modulation properties and potential neuroprotective effects, which are key health concerns for consumers.

## 2. Flavonols

Flavonols are an important subset of natural flavonoids that are widely distributed in various plant species, including fruits, vegetables, nut seeds, spices, herbs, and flowers [5]. Examples of products rich in flavonol content include tea (*Camellia sinensis*), onions (*Allium cepa*), apples (*Malus domestica*), coffee (*Coffea arabica*), tomatoes (*Solanum lycopersicum*), grapes (*Vitis vinifera*), and saffron (*Crocus sativus*) [7]. Research studies often focus on quercetin, kaempferol, and their glycosidic derivatives due to their prevalence in foods. While their presence as aglycones in nature is lower [8], both aglycones and glycosides are found as individual ingredients in many commercial dietary supplements.

**Figure 1:**
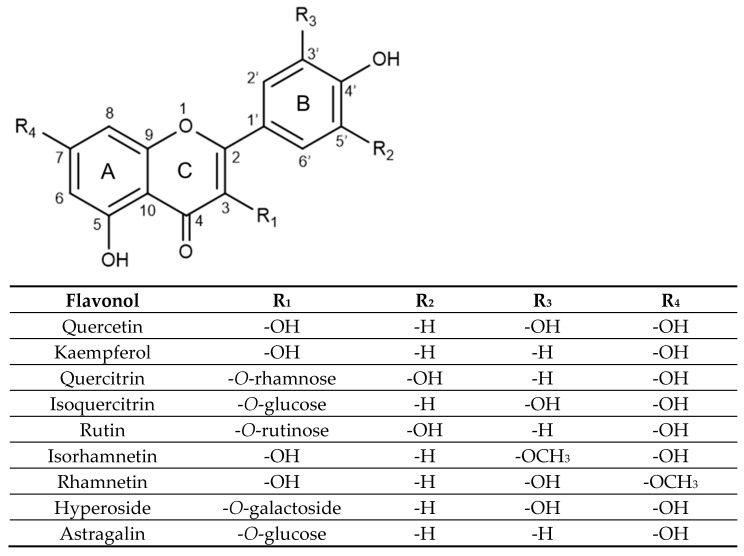
Substitution pattern of selected flavonols and their glycosidic and methoxylated derivatives.

In terms of structure, flavonols are a class of flavonoids with two fused phenyl rings (rings A and C) connected to a heterocyclic ring (ring B), based on the same 3-hydroxyflavone backbone (see Figure 1). Quercetin and kaempferol differ in the placement of hydroxyl groups on ring B, with quercetin having two groups at positions 3′ and 4′, while kaempferol has a single hydroxyl group at position 4′. Within the realm of glycosides, quercitrin, isoquercitrin, rutin, hyperoside, rhamnetin, and isorhamnetin are all derived from a quercetin aglycone structure.

Quercitrin, isoquercitrin, rutin, and hyperoside are all substituted on the 3-position of the C ring, with a glycosidic group replacing the hydroxyl group on the 3-hydroxyflavone base structure. Quercitrin contains a rhamnose sugar, isoquercitrin contains glucose, hyperoside contains galactose and rutin contains a disaccharide rutinose. Rhamnetin and isorhamnetin are both *O*-methylated aglycones, differing only in the methoxy group position. Rhamnetin has its methoxy group at the C7 position on the A ring, while isorhamnetin has its methoxy group at the C3′ position on the B ring. Lastly, astragalin is a kaempferol glycoside with a 3-*O*-glucoside moiety.

### 2.1. Antioxidant Activity

Flavonols have been reported to act as effective antioxidants through various mechanisms such as direct scavenging of ROS, activation of antioxidant enzymes, metal chelation, reduction of α-tocopherol radicals, inhibition of oxidases, mitigation of oxidative stress from nitric oxide, and increase in uric acid levels. However, there is still much discussion about the exact mechanisms that give flavonols these properties [9].

Different studies have suggested that the number of hydroxyl substitutions on the B ring greatly affects antioxidant activities. For example, when comparing Trolox Equivalent Antioxidant Capacity (TEAC) values of kaempferol, which has only one hydroxyl group at C4′, and quercetin, which has a catechol B-ring with *O*-hydroxyl substitutions at C3′ and C4′, the findings indicated a TEAC value of 1.3 mM for kaempferol, much lower than the 4.7 mM value for quercetin. Findings suggest that the number of OH groups has a greater impact on activity than the position. For instance, morin, which has two hydroxyl groups *meta*-substituted at C2′ and C4′, had a TEAC activity of 2.6 mM [10].

The antioxidant activity of kaempferol, quercetin and myricetin was assessed using 2,2-diphenyl-1-picrylhydrazyl (DPPH). The results showed that myricetin, which has three OH groups at C3′, C4′ and C5′, had the highest scavenging activity with the lowest EC_50_ value of 13.77 mg/mL, followed by quercetin at 18.43 mg/mL, and kaempferol at EC_50_ of 27.63 mg/mL, indicating the lowest activity. A similar trend was observed with 2,2-azino-bis-3-ethylbenzothiazoline-6-sulphonic acid (ABTS), where myricetin exhibited the highest antioxidant potential with an EC_50_ of 5.78 mg/mL, while kaempferol had the highest EC_50_ at 17.11 mg/mL, and quercetin had an intermediate value of 7.41 mg/mL.

The Ferric Reducing Antioxidant Power (FRAP) findings also showed similar results expressed as EC1 or the ferric ability equivalent of 1 mM FeSO_4_. Myricetin had the lowest value of 0.15 mM, indicating the best antioxidant potential, followed closely by quercetin at 0.16 m, and kaempferol showing the highest EC1 at 0.27 mM, indicating lower antioxidant activity [11].

Regarding methoxylated derivatives, studies have shown that increased substitution of hydroxyl groups by methoxy groups reduces the antioxidant activity of the flavonol. For instance, a DPPH assessment of the radical scavenging potential of isorhamnetin and tamarixetin, which has methoxy groups at C4′ and C3′ of the quercetin B-ring, showed an IC_50_ value of 1.33 ug/mL for quercetin, while the methoxylated derivative had 2.5-fold higher values, indicating lower antioxidant potential [12].

Additionally, results from a FRAP assay showed a similar trend, with quercetin having a 1.8-fold higher antioxidant value than isorhamnetin and tamarixetin. It is noteworthy that all three flavonols displayed greater antioxidant activity than butylated hydroxytoluene (BHT) used as a standard [12]. Meanwhile, it has been reported that other methoxylated derivatives like rhamnetin, which has a methoxy group at C7 of the A-ring, exhibit similar antioxidant potential when compared to quercetin [13].

Regarding glycosylation, it is suggested that glycosidic forms in the C3 position of the C-ring from flavonols generally have lower in vitro antioxidant activities compared to the aglycones. This is attributed to steric effects that hinder radical resonance stabilization and the relation of a free hydroxyl group in that position with radical resonance stabilization. For example, in vitro experiments involving TEAC assays on quercetin and its glycoside rutin, which contains a rutinoside moiety in C3, reported values decreasing to 2.1 mM from 4.7 mM when comparing rutin to quercetin [10].

Other studies using DPPH and ABTS assays also showed a higher antioxidant potential for the quercetin aglycone, with lower EC_50_ values of 18.43 and 7.41 mg/mL compared to 26.05 and 33.46 mg/mL for rutin. Additionally, FRAP results demonstrated a lower antioxidant value for rutin at EC_1_ = 0.22 mg/mL compared to 0.16 mg/mL for quercetin [11].

Of particular interest in this work is how these effects might overlap or produce other beneficial biological effects, such as inflammation modulation and neuroprotection [9]. In fact, quercetin and kaempferol, along with their glycosides, have been highly sought after for their relative effectiveness in a variety of biological contexts as well as their relative commonness among different sources. Therefore, in the following sections we will discuss their linked anti-inflammatory and neuroprotective potential benefits.

### 2.2. Anti-Inflammatory Effects

Flavonols have been evaluated as a treatment for inflammatory conditions, with several different pathways assessed for their activity. Studies have shown that a 50 µM dose of quercetin has anti-inflammatory effects in Lipopolysaccharide (LPS) stimulated bone-marrow-derived macrophages (BMDM), inhibiting the production of Tumor Necrosis Factor-alpha (TNF-α) and Interleukin-1 beta (IL-1β) inflammatory cytokines. On the other hand, the glycosylated derivative quercitrin did not show inhibitory effects [14].

Similar inhibitory effects on LPS-stimulated RAW 264.7 cells were reported for both quercetin and quercitrin in another study. Both compounds showed inhibition of Nitric Oxide (NO) production, with quercetin requiring a lower concentration (3.5 µg/mL) compared to quercitrin (4.5 µg/mL) [15]. Furthermore, in vivo studies demonstrated that quercitrin (50µM) was hydrolyzed by intestinal bacteria to quercetin, which then exhibited anti-inflammatory effects by inhibiting the production of TNF-α and IL-1β cytokines in colitis-induced Wistar rats [14].

Rutin was found to effectively inhibit in vivo secretion of Interleukin-6 (IL-6) at concentrations below 20 µM, and down-regulate TNF-α and IL-1β at concentrations of 50 µM and >100 µM, respectively [16]. Astragalin was similarly shown to reduce these cytokine levels in LPS-induced acute lung injury (ALI) in BALB/c mice pre-treated with doses of 50 or 75 mg/kg in a dose-dependent manner [17]. Meanwhile, isorhamnetin was found to reduce TNF-α, IL-1β, and IL-6 production in LPS-stimulated RAW 264.7 cells in a dose-dependent manner from 0.25 to 1 nM doses [18] and from 2.5 to 10 µg/mL doses [19]. In vivo experiments showed that doses of 30 and 60 mg/kg of isorhamnetin inhibited TNF-α, IL-6 and IL-1β secretion in mice with LPS-induced acute lung injury, indicating significant effects compared to dexamethasone [19].

The anti-inflammatory activity of rhamnetin was evaluated in LPS-stimulated RAW264.7 cells. Co-treatment of the cells with 10 and 20 μM of rhamnetin was shown to decrease NO production by 62% and 74%, respectively, as well as to downregulate TNF-α production by 54% and 63%, respectively. Furthermore, rhamnetin at 20 μM suppressed the phosphorylation of c-Jun N-terminal kinase (JNK), p38 Mitogen-Activated Protein kinase (p38 MAPK), and Extracellular Signal-Regulated kinase (ERK) by 64%, 51%, and 27%, respectively, and inhibited Cyclooxygenase-2 (COX-2) expression by 76% [20].

These results, along with the lack of toxicity towards normal cells, demonstrate the potential of rhamnetin due to its anti-inflammatory in vitro effects. A novel scaffold, 3-arylisocoumarin with a 3-methoxy-4-hydroxy system in the B-ring similar to isorhamnetin, was synthesized via alkylation of an α-aryl aminonitrile followed by hydrolysis and intramolecular cyclization of the intermediate aryl ketone. The evaluation of the effects of this 3-arylisocoumarin at 50 µM on 5-Lipoxygenase (5-LOX) activity and the level of prostaglandin E_2_ (PGE_2_) in HeLa cervical cancer cells, indicated inhibition of 5-LOX and PGE_2_ production with IC_50_ of 4.6 and 6.3 mM, respectively, thereby demonstrating important anti-inflammatory effects [21].

Another in vitro study compared nineteen flavonoids extracted from *Apocyni Veneti Folium* regarding their inhibitory effect on COX-2 and used biolayer interferometry to confirm affinity. Astragalin, isoquercitrin, and hyperoside exhibited the strongest enzyme inhibition and the highest affinity with COX-2 [22]. In turn, the effects of hyperoside (20 mg/kg every other day) in an osteoarthritis destabilized medial meniscus (DMM) model in C57BL/6 mice were evaluated after 4 and 8 weeks of treatment. Findings indicated progressive superficial cartilage destruction and erosion in the untreated model group, while the hyperoside-treated group exhibited a smoother and more intact cartilage surface, suggesting anti-inflammatory protective effects [23].

Other studies have also investigated the impact of flavonols on the nuclear factor-kappa B (NF-κB) pathway as a potential mechanism for reducing inflammation. For example, one study demonstrated that quercetin, but not quercitrin, inhibited the phosphorylation of IκB-α in LPS-stimulated BMDM macrophages, thereby downregulating the NF-κB pathway. Additionally, in vivo experiments conducted on dextran sulfate sodium (DSS) colitis-induced rats showed that quercitrin released quercetin, leading to a decrease in the levels of activated NF-κB molecules back to normal values [14].

Astragalin has also shown to inhibit the activation of the IκB kinase complex (IKK), thereby downregulating the NF-κB pathway through inhibition of IκBα protein degradation in LPS-induced lung injury BALB/c mice at doses of 50 and 75 mg/kg [17]. A similar study conducted on ALI-induced BALB/c mice using isorhamnetin (6, 12 and 24 nM) indicated a dose-dependent inhibition of IκBα phosphorylation and subsequent downregulation of the NF-κB pathway [18].

A different study examined the impact of quercetin and its glycosides, including rutin, quercitrin, isoquercitrin, and hyperoside, on LPS-induced PGE2 levels in RAW 264.7 cells. The results showed that rutin did not have a significant inhibitory effect. However, quercetin, quercitrin and isoquercitrin at concentrations of 5µM significantly decreased LPS-induced PGE2 levels, by 66%, 60%, and 54%, respectively. Hyperoside required a concentration of 10µM to achieve a 56% reduction, while quercetin exhibited the highest inhibition rate of 96% at a concentration of 40 µM [24].

Hyperoside also significantly reversed the up-regulation of NR2B-containing N-methyl-D-aspartate (NMDA) receptors in the periaqueductal gray (PAG) of mice with induced peripheral injury, which aided in the perception of inflammatory pain [25]. Additionally, quercitrin has shown a decrease in proinflammatory and proatherogenic vascular endothelial growth factors (VEGF) at a dose of 10 μmol/L blocking VEGF expression upregulated by Cu^2+^-oxidized-low-density lipoprotein (Cu^2+^-oxLDL) in J774A1 mouse histiocytic lymphoma cells [26].

### 2.3. Neuroprotective Potential

Flavonols have been shown to have neuroprotective effects that are related to memory deficits. For instance, a study found that quercetin and isorhamnetin glycoside derivatives can inhibit acetylcholinesterase (AChE), which is a therapeutic approach for treating these disorders. Several derivatives exhibited significant AChE inhibition, with isorhamnetin-3-O-(2″,6″-*O*-di-acetyl)-glucoside (IC_50_ 51.25 μM) and quercetin-3-O-(2″,6″-*O*-di-acetyl)-glucoside (IC_50_ 36.47 μM) displaying the highest values. Since the only structural differences between quercetin and isorhamnetin lie in the methoxy group, the study suggested that these inhibitory effects could be attributed to the hydrogen bonds of the hydroxyl group at the C-7 position with Asp74 or Tyr72 residues and those of C-3′ and C-4′ with Ser203 and Gly121 residues as well as the interactions of the C-4-keto group and the residue Phe295 [27].

Another important neuroprotective activity is related to ischemic stroke injuries, as antioxidants can prevent free radical-induced neural damage. For instance, a study on cerebral ischemia–reperfusion (IR) induced in Wistar rats using the middle cerebral artery occlusion (MCAO) model and reperfusion, showed that pre-administration of rutin at a dose of 25 mg/kg significantly restored antioxidant enzymes glutathione peroxidase (GPx), glutathione reductase (GR), catalase (CAT), and superoxide dismutase (SOD) activities. This led to a reduction in infarction size and suppression of neuronal loss [28].

A different study using an intraluminal filament model of MCAO in IR mice showed that isorhamnetin at a dose of 5 mg/kg reduced the production of pro-inflammatory cytokines IL-1β, IL-6 and TNF-α by 40 to 50%, as well as suppressed iNOS expression, contributing to protective effects against induced ischemic stroke [29]. Astragalin has also been found to improve long term neurological outcomes in a cerebral Ischemia/Reperfusion injury intraluminal occlusion model in Male Sprague-Dawley rats, inhibiting production of IL-1β, IL-6, IL-8 and TNF-α in a range between 40 and 60% and suppressing COX-2 and iNOS expression in a range of 30-35% when administered at 50µM doses [30]. In turn, quercitrin reduced infarction volume in mice with ferric chloride (FeCl_3_)-induced thrombosis when treated with 50 mg/kg doses, preventing platelet thrombus formation [31].

Other important neuroprotective effects involve treatments for neurodegenerative diseases, such as Parkinson’s disease (PD) and Alzheimer’s. For example, a study on rotenone PD-induced rats showed that hyperoside at a dose of 200 mg/kg increased the number of Tyrosine hydroxylase (TH)-positive cells and notably inhibited cell apoptosis in substantia nigra par compacta (SNpc) tissues. Additionally, hyperoside 2 µM inhibited the expression of apoptosis-related Bax proteins, cleaved caspase 3 and CyC as well as autophagy-related Beclin 1 and LC3II proteins in a rotenone-injured SH-SY5Y human neuroblastoma-cell model [32].

It is well known that free radicals induce oxidative stress, which is a causal factor in the aforementioned neurodegenerative diseases. To explore the protective effects of rutin and isoquercitrin, a study was conducted using rat pheochromocytoma (PC-12) cells exposed to 6-hydroxydopamine (6-OHDA) model. The results indicated that both flavonoids reduced markers of oxidative stress by inhibiting the production of CAT, GPx, GR, and SOD enzymes. In terms of cell viability, when PC-12 cells were pre-treated with 100 µM of flavonoids, rutin showed the highest protection (78.4% cell viability) after 6-OHDA exposure. When the flavonoids were added concomitantly with 6-OHDA, rutin showed 75% cell viability, while isoquercitrin had a lower effect with a 56.9% cell viability. The study suggests that these results can be attributed to the difference in structure due to the fact that the additional rhamnose glycoside moiety in rutin is associated with higher solubility [33].

Other flavonols have also been implicated in effects that may provide benefits for patients with Alzheimer’s disease (AD), mostly in the prevention or treatment of amyloid plaques. A study on a cell system overexpressing the APP Swedish mutation (APPswe) associated with early-onset familial AD, which produces oxidative stress by enhancing amyloid beta (Aβ) production, showed that quercetin and rutin were able to effectively inhibit the formation of Aβ fibrils and disaggregate Aβ fibrils. Quercetin was more effective at preventing aggregation at 1 µM, but both flavonols had similar effects above 5 µM. Additionally, they both exhibited similar disaggregation activities and were dose-dependent. The authors also studied the activity of β-secretase enzyme (BACE) and found that it was significantly inhibited by 100 µM rutin. Furthermore, when APPswe cells were treated with H_2_O_2,_ quercetin and rutin almost completely inhibited ROS generation. These findings are consistent with other results suggesting that hydroxyphenyl ring structures can inhibit fibril formation and destabilize preformed fibrils [34].

Quercitrin also exhibited positive effects, with concentrations above 50 µM attenuating Aβ_25–35_ induced neurotoxicity and increasing cell viability from approximately 55% to over 70% [35]. Hyperoside has also been discussed as a potential treatment for AD protection. A study found that pre-treatment with concentrations of 5 µM, 10 µM, and 20 µM led to a dose-dependent decrease in nuclear condensation and fragmented morphology induced by Aβ_25–35_ [36]. Similarly, a study on astragalin revealed that its administration suppressed Aβ_1–40_ and Aβ_1–42_ deposition, tau hyper-phosphorylation, and neuronal cell death in SAMP8 mice and Aβ_1–42_O-treated neurons. Additionally, it upregulated the expression of estrogen receptor alpha (Erα) and estrogen receptor beta (Erβ) in the hippocampus of the mice [37].

Similarly, isorhamnetin protected against Aβ-induced cytotoxicity by destabilizing Aβ aggregates at concentrations as low as 3 µM [38]. A novel scaffold, 8-hydroxy-3-arylisocoumarin, with a 5-OH in ring A and an OCH_3_ group in ring B, was evaluated as potential agonist of Tropomyosin receptor kinase B (TrkB), the main receptor for Brain-Derived Neurotrophic Factor (BDNF) in transiently transfected human embryonic kidney HEK293T cells. The results indicated a high affinity of the arylisocoumarin with TrkB, showing an EC_50_ of <90 nM. Furthermore, the study suggested that it exhibited a neurotrophic action similar to BDNF through the activation of the TrkB receptor, demonstrating its potential as TrkB agonist. This suggests neuroprotective effects against cognitive decline and neurodegenerative diseases like Alzheimer’s [39].

Flavonols have also been implicated in the treatment of other neurological disorders. Epilepsy is one such condition where it was found that a pre-treatment dose of 50 mg/kg of hyperoside could help prevent epilepsy-induced neuronal damage in the hippocampal CA3 region of ICR mice [40]. Schizophrenia is another condition in which some flavonols have been studied, such as rutin, which was shown to significantly reduce stereotypical schizophrenic behavior in ICR mice at a dose of 0.1 mg/kg, suggesting rutin’s potential effectiveness in alleviating symptoms of schizophrenia at a specific dose [41]. These studies demonstrate the diverse potential neuroprotective effects of flavonol aglycones and their glycosidic derivatives.

## 3. Flavones

Flavones are a class of flavonoids with a 2-phenylchromen-4-one backbone. These compounds are relatively abundant in green vegetables, such as luteolin in celery (*Apium graveolens*), and in herbs, for instance, mainly apigenin and its *O*-glycosides in parsley (*Petroselinum crispum*), as well as apigenin and luteolin *O*-glycosides in oregano (*Origanum vulgare*) and basil (*Ocimum basilicum*), including holy basil or tulsi (*Ocimum sanctum* or *Ocimum tenuiflorum*). Tea ingredients, such as chamomile (*Matricaria chamomile*), are also rich in apigenin *O*-glycosides, while green and black teas have apigenin and luteolin *C*-glycosides derivates [42]. Meanwhile, passionflower (*Passiflora edulis* and *Passiflora incarnata*) has attracted much attention as a dietary supplement because of its rich (iso)vitexin and (iso)orientin C-glycosides content and potential health benefits.

**Figure 2:**
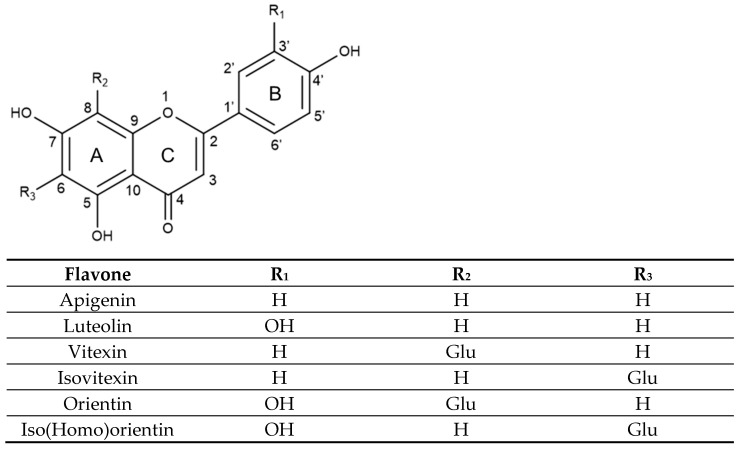
Substitution pattern of selected flavones and their glycosidic derivatives.

Flavones hold a double bond between C2 and C3 in their backbone structure, no substitution at the C3 position, and they are oxidized, holding a carbonyl at the C4 position in ring C [42], as seen in Figure 2. For instance, apigenin and luteolin differ only in the hydroxy substituents in ring B, with apigenin holding a single OH group in C4′ while luteolin has an additional hydroxyl group in C3′.

Vitexin and isovitexin are C-glucoside derivatives of apigenin, each containing one hydroxyl group at C4′ of the B aromatic ring. However, their chemical structure differs in the position of the glucose moiety at C8 or C6 of the A aromatic ring, respectively. On the other hand, orientin and isoorientin, also called homoorientin are C-glucoside derivatives of luteolin, holding two hydroxyl groups in C3′ and C4′ of the B aromatic ring and also differing in the glycosylation at C8 or C6 of the A aromatic ring, respectively.

### 3.1. Antioxidant Activity

Theoretical studies on the structure–activity relationship analysis of flavones regarding their antioxidant potential have highlighted the importance of the 3′-OH functional group in the B ring and the *C*-glycosylation in the A ring. For example, the *o*-di-hydroxyl structure in the B-ring has been shown to contribute to effective radical scavenging in luteolin compared to apigenin. This is due to the lesser A-ring radical species contribution as indicated by its higher bond dissociation energy (BDE) value. The 1,4-pyrone structure with a 2,3-double bond and the C4-oxo group acts as a strong electron-withdrawing moiety, reducing its scavenging activity [43,44].

In vitro studies assessing the antioxidant activity of these flavones support the significance of the catechol B-ring moiety in the formation of stable radical species. For instance, luteolin demonstrated a better Trolox equivalent (TE) value of 2.18 mM compared to apigenin with 0.086 mM in an ABTS^•+^ assay. A similar trend was observed in a DPPH assay, with luteolin showing a higher TE value of 2.24 mM compared to 0.041 mM of apigenin [45]. Additionally, results from another study using the FRAP assay showed a higher antioxidant value of 2.23 mM Fe (II) for luteolin, while apigenin exhibited a 2.01 mM Fe (II) value [46].

In addition, studies using density functional theory (DFT) have indicated that a lower BDE value was obtained when a C-glycoside was present in ring A, associated with higher hydrogen-donating ability of the B-ring, therefore enhancing their antioxidant activity compared to the aglycone [43,47]. Various in vitro studies assessing aglycones and their C-glycosides support these theoretical assertions. For example, a study on apigenin and its C8 glycoside vitexin using the DPPH assay reported that vitexin had a TE value of 0.205 mM, five times higher than the value obtained for apigenin. Similarly, the ABTS^•+^ results showed a TE value of 0.216 mM for luteolin, while apigenin had a 2.5-fold lower value [45]. Other findings also indicate a similar trend using FRAP assay comparing luteolin and its C8 glycoside orientin, with the latter showing a Fe (II) value of 2.81 mM, higher than the 2.23 mM obtained for luteolin [46,48].

Comparative studies related to the position of C-glycosylation in ring A have also been conducted. For example, comparing the C6 glycosylated apigenin and luteolin derivates, isovitexin and isoorientin, respectively, and their C8 glycosylated derivates, vitexin and orientin. The results have shown that C-6 glycosylation increases the antioxidant capacity of the molecule by enhancing radical stabilization. A study using the FRAP methodology showed a Fe (II) value of 1.0 mM for isovitexin, which was 1.8 times the value obtained for vitexin [46]. Another study dealing with ABTS^•+^ compared the antioxidant capacity of the four C-glycosylated flavones, indicating that isoorientin showed the lowest IC_50_ value of 11.25 µM TE, followed by orientin with an 11.43 µM TE, thereby exhibiting much higher antioxidant activity than isovitexin and vitexin that showed values of 1224 µM TE and 2313 µM TE, respectively [49].

These results show C-6 glycosides have higher radical scavenging activity than C-8 glycosides in ring A, and also highlight the importance of the 3′ and 4′-OH substitution in ring B, as addressed in other studies. For instance, using the TEAC assay, it was found that isoorientin and orientin had the highest values of 1.88 mM and 1.56 mM TE, respectively. Meanwhile, isovitexin and vitexin showed much lower activity with values of 0.14 mM and 0.02 mM TE, respectively. Additionally, a DPPH assay resulted in a similar trend, with isoorientin and orienting holding 2.88 mM and 2.38 mM TE values while isovitexin and vitexin exhibited 0.007 mM and 0.006 mM TE values [50].

Another study using FRAP and DPPH assays also reported that isoorientin showed the highest FRAP value (947.15 mg TE/g) compared to orientin, which had a value of 783.11 mg TE/g. Meanwhile, isovitexin and vitexin had much lower values of 129.12 and 116.68 mg TE/g, respectively. DPPH assay results indicated a similar trend, with isoorientin having the lowest IC_50_ (0.08 mg/mL) followed by orientin (0.09 mg/mL), while isovitexin and vitexin showed much higher IC_50_ values of 0.37 and 0.41 mg/mL, respectively [51,52]. Overall, these results suggest the antioxidant potential of these different aglycone and glycosylated flavones, which are linked to their anti-inflammatory and neuroprotective potential benefits, discussed in the following sections.

### 3.2. Anti-Inflammatory Effects

A study on the anti-inflammatory effects of apigenin in LPS-activated mouse macrophage RAW264.7 cells, showed that this flavone inhibited PGE_2_ and NO expressed as nitrite with an IC_50_ of 8.04 µM and 9.86 µM, respectively. Additionally, apigenin at 15 µM suppressed COX-2 and inducible nitric oxide synthase (iNOS) protein expression levels by more than 50%. The study also indicated that the inhibition was mostly regulated at the transcription level and apigenin almost completely suppressed the seven-fold increase in NF-κB transcription factor induced by LPS treatment in RAW264.7 cells [53,54].

Another study on Bovine Serum Albumin Advanced Glycation End products (BSA-AGE)-induced NO and TNF-α production showed that apigenin inhibited both pro-inflammatory factors in a dose-dependent manner, with EC_50_ values of approximately 14 μM and 8 μM, respectively [55]. In turn, an LPS-treated N-11 murine microglia model was used to study the anti-inflammatory effect of apigenin in respect to NO production, reporting an IC_50_ of 15 µM. Furthermore, apigenin showed an even higher potential for inhibiting NO when applied as a pre-treatment to LPS-activated RAW264.7 cells, exhibiting an IC_50_ of 7 µM and was able to inhibit TNF-α pro-inflammatory cytokine production with an IC_50_ of 5 µg/mL [56].

The LPS-activated macrophage RAW 264.7 cells model was used to assess the anti-inflammatory activity of apigenin and luteolin. The findings reported that both flavones inhibit iNOS activity with IC_50_ values of 23 µM and 27 µM, respectively. Meanwhile. flavanones and other flavonoid glycosides did not show significant inhibition, indicating the importance of the C-2,3 double bond and substitution patterns on the inhibition strength [57]. Another study using a similar model of LPS-treated RAW 264.7 cells showed a 12.5% increase in NO concentration, which was reversed by pre-treatment with apigenin or luteolin at 200 µM, restoring NO levels to normal. Additionally, the study found that LPS-induced phagocytic activity, which had increased by 28%, was suppressed by apigenin by 37.5% and luteolin by 50% [58].

It was also reported that 10 mg/kg of vitexin was able to treat inflammatory pain through the modulation of cytokine production in mice with induced carrageenan paw inflammation. This included the inhibition of pro-inflammatory cytokines TNF-α, IL-1β, IL-6, and IL-33, as well as the enhancement of the anti-inflammatory cytokine IL-10. The largest inhibition value of approximately 80% was found on IL-1β followed by an inhibition effect of around 45% on TNF-α while IL-10 production increased by 75%. The inhibition of the other two cytokines IL-6 and IL-33 showed a value of approximately 35% [59].

The anti-inflammatory activity of isovitexin was assessed in LPS-stimulated RAW 264.7 cells. Findings from one study indicated inhibition of TNF-α secretion with an IC_50_ of 78.6 μM, PGE2 formation with an IC_50_ of 80.0 μM, and downregulation of COX-2 expression by 58% [60]. Another study showed the suppression of NO overproduction with an IC_50_ of 58.5 μM as well as prevention of NF-*κ*B translocation [61]. In turn, pre-treatment with orientin in RAW 267.4 cells alleviated LPS-induced inflammatory responses. Specifically, orientin inhibited the enhancement of IL-6, TNF-α, and IL-1β pro-inflammatory cytokines secretion by approximately 55%, 65% and 70%, respectively. It also reduced the phosphorylation and degradation of I*κ*B*α* as well as the nuclear translocation of NF-*κ*B [62].

Isoorientin and orientin were found to reduce the production of ROS with IC_50_ values of 0.16, and 0.15 μM, respectively, in human neutrophils treated with N-formylmethionyl-leucyl-phenylalanine (fMLP). They also inhibited the formation of neutrophil extracellular traps (NETs) induced by treatment of the neutrophils with phorbol 12-myristate 13-acetate (PMA). Isoorientin at a concentration of 30 µM produced a 30% inhibition level, while orientin at the same concentration exerted a higher 40% inhibition value. These results are significant because elevated NET levels have been linked to increased proinflammatory responses in SARS-CoV-2 and other respiratory viruses [63].

### 3.3. Neuroprotective Potential

Flavones have shown neuroprotective effects in treating cognitive and neurological conditions. For example, in a study on cerebral ischemia/reperfusion (I/R), the effects of apigenin were evaluated in PC12 rat adrenal pheochromocytoma cells treated with cobalt chloride to induce an oxidative stress injury model. The findings reported that pre-treatment with 10 mg/mL of apigenin increased cell viability to 73.78%, reduced ROS levels by 65%, and showed a three-fold increase in mitochondrial membrane potential (MMP) dissipation. Another study using middle cerebral artery occlusion (MCAO) in adult male Sprague Dawley rats indicated that treatment with apigenin at a dose of 25 mg/kg resulted in a 23.5% reduction in infarct volume and improved neurological deficit scores in MCAO rats [64,65].

The neuroprotective effects of apigenin against I/R were assessed using an in vitro LPS-stimulated murine microglia BV-2 cells model and an in vivo MCAO-induced model in adult male ICR mice. The results showed that apigenin inhibited LPS-induced NO production in a dose-dependent manner, achieving a 45% reduction at 5 µM and nearly 80% reduction at 10 µM, while luteolin also tested in this assay, resulted in a 15% and 45% reduction at similar concentrations. Furthermore, apigenin at 10 µM inhibited PGE2 production by 70% by suppressing COX-2 protein expression. In the in vivo model, apigenin at 20 mg/kg reduced the infarct volume by 70% compared to the effect achieved by 3-methyl-1-phenyl-2-pyrazolium-5-one (MCI-186) used as a positive control. Additionally, apigenin decreased microglial activation by 30% in MCAO-induced ICR mice [66].

Another study involving male Kunming MCAO-induced mice showed that vitexin reduced infarct volume in a dose-dependent manner at doses of 3.25, 7.5 and 15 mg/kg, achieving a 90% reduction at the highest dose. This result was similar to the one obtained with breviscapine, used as a positive control. Additionally, vitexin improved neurological deficits, with I/R mice deficit scores increasing by 60% at the 15 mg/kg dose compared, representing a 10% higher value than the improvement seen with breviscapine. Vitexin was also found to downregulate p-JNK and p-38, as well as to increase Bcl-2 expression in the hippocampus and cortex. At the 15 mg/kg dose, vitexin had a similar effect to breviscapine, suggesting that vitexin may alter the MAPK signaling pathway [67].

A study conducted on embryonic day 17 (E17) and 18 (E18) Sprague-Dawley rat embryos using oxygen-glucose deprivation/reperfusion (OGD/RP)-induced cytotoxicity revealed that orientin could reverse the cytotoxic effects in a dose-dependent manner. At 30 µM, orientin had a similar effect to nimodipine, which was used as a positive control. Furthermore, pre-treatment with orientin at 30 µM stabilized OGD/RP-induced dissipation of MMP, downregulated caspase-3 activity, and reversed the decrease in Bcl-2 protein expression. The study suggested that the neuroprotective effects were mediated through the JNK and ERK1/2 signaling pathways [68].

In a separate model involving MCAO in adult male Sprague-Dawley rats, it was observed that orientin at a dose of 6.48 µmol/kg alleviated neurological impairment effects to a similar extent as edaravone (3.24 µmol/kg) used as a positive control. This was evidenced by a reduction in infarct volume, decreased cerebral edema, increased SOD and ATP synthase (ATPase) activities, and a decrease in malondialdehyde (MDA) content [69].

Studies on protective effects related to neurodegenerative diseases such as Parkinson’s (PD) and Alzheimer’s disease (AD) are often performed in relation to microglia-mediated inflammation. For example, a PD model induced by 1-methyl-4-phenyl-1,2,3,6-tetrahydropyridine (MPTP) in C57BL/6 mice showed that administering apigenin at a dose of 15 mg/kg reversed the changes in IL-6, TNF-α, and IL-1β, reducing their secretion by 27.3%, 29.8%, and 39.0%, respectively, while increasing IL-10 production by 58.1% and tyrosine hydroxylase (TH) immunopositive neurons by 37.1% [70]. Similarly, findings in a different PD model involving rotenone-induced toxicity in microglial BV2 cells, showed that pre-treatment with luteolin at 1 µM increased cell viability from 40% to 80%, decreased IL-1β by 70% and regulated oxidative stress, thus protecting microglia [71].

In vitro treatment with methyl-4-phenylpyridine (MPP^+^) on human neuroblastoma SH-SY5Y cells and in vivo MPTP treatment in C57BL/6 mice used as PD models demonstrated the neuroprotective effects of vitexin. For instance, vitexin (10–40 µM) protected cell viability in a dose-dependent manner, reaching 90% at 40 µM while treatment with vitexin (50 mg/kg) prevented bradykinesia and alleviated initial lesions. Results suggested that the suppression of MPP^+^-induced apoptosis and protection of dopaminergic neurons were achieved via the PI3K/Akt signaling pathway [72].

The neurodegenerative effects induced by rotenone in SH-SY5Y cells and in Swiss albino mice PD models were mitigated by orientin. Orientin downregulated oxidative stress and inflammatory markers, modulated gene expression and improved behavioral parameters. Pre-treatment of SH-SY5Y cells with 20 µM vitexin reduced ROS production by 50% and restored CAT, SOD, and GPx activities to control levels. Similar results were observed with a dose of 20 mg/kg vitexin in mice, where rotenone-induced motor impairment parameters were also alleviated [73].

A study on Alzheimer’s disease (AD) showed that apigenin and luteolin can decrease CD40 expression on the surface of microglial cells by investigating the upstream signal transducer and activator of transcription (STAT1) signaling pathway. When N9 and primary derived cell lines were treated with a 25 μM concentration of either apigenin or luteolin in the presence of IFN-γ, findings indicated suppression of microglial TNF-α and IL-6 inflammatory cytokines production and inhibition of IFN-γ-induced phosphorylation of STAT1 [74].

Another study evaluated apigenin along with its C-glycosides vitexin and isovitexin in relation to potential protective effects against Alzheimer’s Disease (AD). This evaluation was based on their inhibitory activity against AChE, butyryl cholinesterase (BChE), and beta secretase 1 (BACE1) enzymes. The three compounds showed inhibition of AChE, with apigenin having the lowest activity (IC_50_ = 34.43 μM), isovitexin holding the best IC_50_ value (6.24 μM), and vitexin exhibiting an intermediate IC_50_ value of 12.16 μM. Isovitexin and vitexin both exhibited higher inhibition against BChE, with IC_50_ values of 6.48 μM and 6.73 μM, respectively, while apigenin had an IC_50_ of 29.11 μM. BACE1 was only inhibited by vitexin (IC_50_ = 51.07 μM). These results indicate the impact of C-glycosylation and its location on the strength and selectivity of inhibition [75].

The neuroprotective effects of orientin were studied in an Aβ_1–42_-induced C57B/6 mouse model of AD. The study showed that a dose of 5 mg/kg was able to decrease levels of ROS, protein oxidation (3-NT), lipid peroxidation (4-HNE), and DNA oxidation (8-OHdG) in the hippocampus by 40%, 60%, 50% and 55%, respectively. Additionally, orientin ameliorated cognitive deficits in Aβ_1–42_-induced AD mice [76].

Vitexin has been shown to provide anti-convulsant and anti-epileptic effects. A study indicated that vitexin may function as a benzodiazepine receptor ligand by delaying the onset of pentylenetetrazole (PTZ)-induced minima clonic seizures (MCS) and generalized tonic–clonic seizures (GTCS) in male Wistar rats. Specifically, when administered at a concentration of 100 μM, vitexin was able to delay the onset of MCS and GTCS by 200 s and 369 s, respectively. This effect is believed to occur through a GABA_A_-benzodiazepine receptor complex, as evidenced by its significant anti-convulsant response [77]. These findings collectively suggest the potential of flavones as neuroprotective agents, although further studies are needed to explore their potential application in the treatment of neurodegenerative diseases.

## 4. Proanthocyanidins

Proanthocyanidins can be found as important components in different plants. Grape seeds and skin are among their richest sources (~30% and 15%, respectively) [78]. Some other common plant sources rich in proanthocyanidins are peanuts (*Arachis hypogaea*) and cocoa (*Theobroma cacao*) [79], persimmons (*Diospyros virginiana*) [80], American wintergreen *(Gaultheria procumbens)* [81], milky iris (*Iris lactea)* [82], sorrel (*Rumex acetosa)* [83], and golden root (*Rhodiola rosea)* [84]. Another great source of proanthocyanidins is tea, with green tea extracts being rich in (−)-epigallocatechin gallate (EGCG), providing almost 7830 mg/100 g of dry green tea leaves [85,86]. Nuts like hazelnuts (*Corylus* spp.), pecans (*Carya illinoinensis*), and carob (*Ceratonia siliqua*) are also good sources of EGCG [87]. Finally, berries such as cranberries (*Vaccinium* spp.) and alpine currants (*Ribes alpinum*) are also a rich source of proanthocyanidins [79]. The content of proanthocyanidins in these natural sources has led to a significant increase in their consumption as dietary supplements.

**Figure 3:**
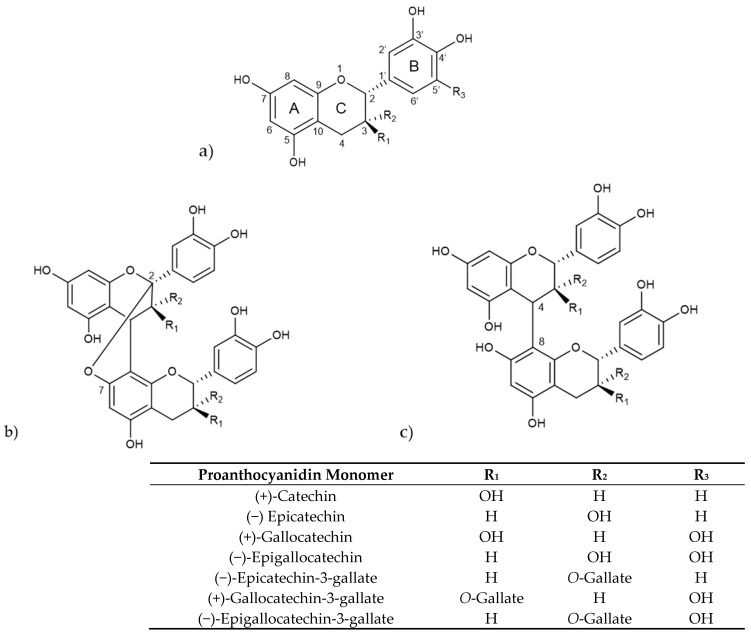
(**a**) Structure of selected proanthocyanidin monomers, (**b**) structure of type-A procyanidin dimer (R_1_ = H, R_2_ = OH or R_1_ = OH, R_2_ = H), (**c**) structure of type-B procyanidin dimer (R_1_ = H, R_2_ = OH or R_1_ = OH, R_2_ = H).

Proanthocyanidins consist of oligomers and polymers of flavan-3-ol units and are frequently referred to as condensed tannins. The various types of proanthocyanidins are primarily determined by the structural units or monomers of flavan-3-ols, the type of linkage, degree of polymerization, spatial arrangement, and the position and nature of any substituent group [88]. The main flavan-3-ol monomers include (+)-catechin, (−)-epicatechin (EC), (+)-gallocatechin (GC), and (−)-epigallocatechin (EGC) [89]. Another significant group of flavan-3-ols consists of these monomers esterified with a gallic acid moiety, leading to galloyl derivatives, like (+)-gallocatechin gallate (GCG), (−)-epicatechin gallate (ECG), and (−)-epigallocatechin gallate (EGCG) (Figure 3).

Procyanidins, a key subgroup of proanthocyanidins, are oligomers and polymers of catechin and epicatechin, while prodelphidins are composed of (epi)-gallocatechins [90]. They form B-type proanthocyanidins when there is a C-C covalent bond between monomer units, primarily at C4–C8, whereas A-type proanthocyanidins are formed when there is an additional ether bond, mainly at C2-O-C7 [91]. Depending on the number of monomer units involved, proanthocyanidins are classified as oligomers holding two to four monomeric units, including dimers, trimers and tetramers, and polymers that hold five or more monomeric units [92].

### 4.1. Antioxidant Activity

Studies have shown a structural-activity relationship between flavan-3-ols and their antioxidant potential, which can vary depending on the extract and the characteristics of the antioxidant assay. For example, a study on seven different fractions of Grape seed proanthocyanidin extract (GSPE) with a mean degree of polymerization (mDP) ranging from 1.35 to 6.97, found that a lower mDP correlated with an increase in antioxidant activity for FRAP and ABTS assays. However, no definitive correlation was found when using the DPPH assay. The proanthocyanidin fraction with an mDP of 2.46 had the highest FRAP value of 6.39 mg Fe(II)/g, while the fraction with an mDP of 1.35 had the lowest ABTS IC_50_ value of 26.73 mg/L indicating higher antioxidant potential [93].

Similarly, a study on Peanut skin proanthocyanidin extracts (PSE) with different mDP levels showed higher DPPH scavenging activity (85.8%) when the mDP was 2.87 compared to an antioxidant value of 78.1% obtained when the mDP was 6.74 [94]. In another study, thirteen fractions of mangrove (*Ceriops tagal*) proanthocyanidin extract were examined. These fractions were primarily composed of B-type procyanidins with an mDP ranging from 1.43 to 31.77. This study revealed a positive correlation between mDP and measured antioxidant values using DPPH and FRAP assays up to an mDP of approximately 10. The fraction with an mDP of 10.56 exhibited maximum values of EC_50_ = 78.13 mg/mL and 5.87 mmol ascorbic acid equivalents (AAE)/g for DPPH and FRAP, respectively. However, fractions with higher mDP did not show a correlation, and their values were similar to those fractions with lower mDP (3.52), approximately 95 mg/L and 4 mmol AAE/g for DPPH and FRAP, respectively [95].

Regarding monomeric units, studies have reported that EC and EGC monomers exhibit lower antioxidant capacity than their gallate ester monomers ECG and EGCG. For example, a comparative study on these four monomers, indicated that EGCG had the strongest DPPH antioxidant activity with the lowest IC_50_ value (1.95 µg/mL) followed by ECG with an IC_50_ of 2.44 µg/mL. FRAP results also showed the highest antioxidant value (0.1015 mmol Fe^2+^) for EGCG with the other gallate ester ECG holding a close value (0.0963 mmol Fe^2+^) [96].

Another comparative study indicated that gallate esters ECG and EGCG exhibited higher antioxidant capacity than EC and EGC using an ABTS assay, with results expressed as Trolox equivalents (TE) showing values of 7.80 and 5.63 TE/mol for ECG and EGCG, respectively. This aligns with FRAP results indicating an antioxidant activity of 2.33 and 2.21 TE/mol, respectively, representing values two-fold higher than those obtained for EC and EGC [97]. The higher antioxidant values of gallate esters confirm the contribution from the gallic ester moiety and in the case of EGCG the additional contribution of the hydroxyl in C5′ in the B ring, making them more potent free radical scavengers.

Considering the monomeric structure, a study on *Uncaria tomentosa* extracts, rich in various types of proanthocyanidins, showed that fractions containing higher levels of procyanidins (PC) exhibited better antioxidant capacity compared to fractions with higher levels of phenylpropanoid substituted derivatives at C7 and C8 of ring A, also known as flavanolignans, which exhibited lower antioxidant activity. For example, the fraction with the highest content of PC and propelargonidins (PP) had an ORAC value of 11.28 mmol TE/g extract, while the fraction with the highest content of flavanolignans had a 35% lower ORAC value. The study also found a positive correlation between ORAC antioxidant values and fractions rich in PP (*r* = 0.895, *p* < 0.05) as well as with PC B2 and B4 dimers (*r* = 0.998, *p* < 0.05). These results suggest that the aromatic monomeric unit bonds and the hydroxy substitution in the A and B rings of these molecules play a significant role in their reactions with reactive oxygen species and therefore in their antioxidant effects [98].

Another study found that PC C1 trimer exhibited superior antioxidant activity compared to catechin, with DPPH scavenging activity IC_50_ values of 5.6 µM and 14.3 µM, respectively. Similarly, it showed better results in HO^●^ scavenging activity with IC_50_ values of 250 µM and 693 µM, respectively. These values were superior to those of Trolox, which was used as a standard [99]. The anti-inflammatory and neuroprotective effects of these flavan-3-ols, often linked to their antioxidant properties, will be discussed in the following sections.

### 4.2. Anti-Inflammatory Effects

Flavan-3-ols have demonstrated anti-inflammatory properties by inhibiting various factors, including proinflammatory cytokines. A study used grape pomace extract rich in procyanidins with mDP values ranging between 2.4 and 13.2, and alpine currant extract rich in procyanidins with mDP values ranging between 3.2 and 12.1. This study reported the inhibition of IL-6 production in LPS-activated murine RAW 264.7 cells. Grape procyanidins with medium mDP ranging between 5.3 and 9.6 exhibited the greatest inhibitory effect at a concentration of 15 µg/mL, similar to alpine currant prodelphinidins with mDP ranging between 6.3 and 9.4. This inhibitory effect was higher than those with mDP < 5.2 and mDP 9.7 or higher. At a concentration of 7.8µM, medium mDP values showed the most inhibition [79].

The importance of the degree of polymerization is further emphasized in another study that assessed the impact of monomers, oligomeric and polymeric proanthocyanidins (PACs) from Persimmon peel extract on Streptozotocin (STZ)-induced diabetic rats. The results indicated that oligomeric PAC with an mDP of 3.3 at doses of 10 mg/kg exhibited the most significant inhibitory effects, reducing iNOS and COX-2 expression levels in the kidneys of diabetic rats by approximately 64% and 48%, respectively. In comparison, similar doses of polymeric proanthocyanidins, only achieved 39% and 11% inhibition, respectively. Furthermore, proanthocyanidins were shown to decrease the elevated levels of NF-*κ*Bp65 protein in STZ-induced diabetic rats, with oligomeric PAC demonstrating the highest inhibition rate of around 50% [80].

Regarding individual molecules, in a study comparing the effects of EC, ECG, EGC, and EGCG on LPS-stimulated RAW 264.7 murine macrophage cells, it was found that all four compounds inhibited the increased production of NO at 50µM. ECG and EGC showed a 30% inhibition, followed by EGCG with a 20% decrease in NO, and EGC with a 12.5% NO inhibitory activity [96]. In another study using LPS-stimulated RAW 264.7 cells, the same four proanthocyanidins and gallocatechin (GC) were tested. The results showed that all compounds inhibited the elevated NO content caused by LPS. The highest inhibition was seen with a simultaneous co-treatment of EGCG and GC at a ratio of 6.5 µg/mL and 3.5 µg/mL. This combination demonstrated 1.5 and 1.6-fold higher NO inhibitory activity compared to individual treatments with 10 µg/mL of each compound [100].

A different study focused on the effects of teas and their main proanthocyanidins (EC, EGC, ECG and EGCG) in LPS-treated rat pulmonary macrophage NR8383 cells. When the cells were treated with 0.5 mg/mL of tea extracts and individual compounds, black tea showed higher iNOS inhibition with an IC_50_ of 0.24 mg/mL compared to green tea (IC_50_ = 0.47 mg/mL). Among individual compounds, ECG showed the highest iNOS inhibition (IC_50_ = 0.41 mg/mL) followed by EGCG (IC_50_ = 0.93 mg/mL). Additionally, EGCG almost completely blocked the induction of nitrite/nitrate and iNOS in the cell lines, suggesting that the gallate structure may play a critical role in these anti-inflammatory mechanisms [101].

On the other hand, a study on eleven isolated procyanidins from peanut skin extract showed that A-type dimers and trimers at a concentration of 50µM exhibit significant inhibition in the production of inflammatory cytokines IL-6 and TNF-α in LPS-stimulated cultured Human Myeloma THP-1 cells. Specifically, the (4β→6)-[epicatechin-(4β→8)]-catechin trimer demonstrated the highest inhibitory effect of 40% and 70%, respectively. This last value is twice as high compared to the other ten studied procyanidins [102].

Finally, another study on the anti-inflammatory potential of procyanidin A and B dimers and trimers from a *Gaultheria procumbens* stems extract in concentrations ranging from 25 to 150 µg/mL, reported a reduction in cytokines TNF-α, IL-8, and IL-1B expression in LPS-stimulated human neutrophils. The results were particularly significant for IL-1B, with a 44.3% inhibition at 25 µg/mL and 87.4% at 150 µg/mL, while the inhibition of TNF-α production reached a value of 57.7% [81].

### 4.3. Neuroprotective Potential

The accumulation of macromolecular damage caused by excessive ROS production related to neurodegeneration can lead to necrosis. Therefore, the use of antioxidants is one of the most researched therapeutic approaches for neurodegenerative diseases. For instance, a study on H_2_O_2_-treated rat pheochromocytoma (PC12) cells showed that proanthocyanidins (PACs) at 4 µg/mL reduced ROS and malondialdehyde (MDA) levels by 23% and 26%, respectively, while increasing SOD, CAT, and GSH-Px antioxidant enzymes production by 46%, 50% and 53%. Additionally, PACs provided neuroprotection in H_2_O_2_-treated zebrafish by downregulating the Nuclear Factor-Erythroid 2-related Factor 2/Antioxidant Response Element (Nrf2/ARE) pathway [103].

A similar study compared the effects of PAC monomers (catechin, EC and ECG), procyanidin dimers (B1, B2, B3, B1-3-*O*-Gallate, B2-3-*O*-Gallate), and procyanidin trimer (C1) at 5 µM on H_2_O_2_-treated PC12 cells and at 25 µM doses on H_2_O_2_-treated zebrafish models. The findings showed that the trimer (C1) exhibited the greatest effects in all aspects, including ROS and MDA decrease by 37% and 39%, respectively. Additionally, it achieved GSH-Px, CAT, and SOD upregulation in a 75–80% range. These results were followed by the dimer group and then at a much lower level by the monomers, pointing to a correlation between the degree of PAC polymerization and neuroprotective effects [104].

An ischemia/reperfusion model consisting of middle cerebral artery occlusion (MCAO) in C57BL/6 mice was used to evaluate the neuroprotective effects of GSPE. The findings indicated a dose-dependent inhibition of MCAO-induced infarct volume at doses of 125, 250 and 500 mg GSPE/kg, with a 50% reduction achieved at 250 mg/kg. Furthermore, this dose of GSPE downregulated BAX and caspase-3 protein expression by 35% and 50%, respectively, and increased Bcl-2 production four-fold compared to MCAO mice levels. These changes protected against altered apoptosis-associated gene expression and improved long-term neurological outcomes in a dose-dependent manner in ischemic stroke MCAO mice [105].

Studies have shown that proanthocyanidins can be an important therapeutic target in the treatment of Parkinson’s disease (PD). A study on a rotenone model of PD in SH-SY5Y dopaminergic cells found that treatment with a proanthocyanidin extract from *Moringa oleifera* seeds (12 µg/mL) significantly decreased rotenone-induced oxidative stress, blocked caspase activation and suppressed apoptosis by inhibiting the p38 MAPK, JNK, and ERK signaling pathways [106]. This is supported by another study that used a 1-methyl-4-phenyl-1,2,3,6-tetrahydropyridine (MPTP)-induced PD model in vitro in PC12 cells and in vivo in C57BL/6 mice. The findings indicated that treatment with an oligomeric procyanidin extract at 5 μg/mL in PC-12 cells and at a dose of 300 mg/kg in mice showed a decrease in ROS production and prevented apoptosis in a range of 65–70% in both MPTP-induced models, downregulating the JNK pathway [107].

Another study was conducted to explore therapeutic treatments for tauopathy, and tau-mediated neurodegenerative diseases using a transgenic JNPL3 mice model. The study focused on the effects of GSPE, which consisted of 75% oligomers with procyanidin B dimers being the most predominant form, followed by 17% polymers, and 8% catechin and EC monomers. The findings revealed that when GSPE was administered at a dose of 150 mg/kg/day for a six-month period, a 90% reduction in neurons with tau-hyperphosphorylation inhibition was achieved [108].

In agreement with these findings, another study reported that pre-treatment with GSPE (100 μg/mL) in Aβ_25–35_-treated PC12 cells, reduced the cell apoptotic rate by 25%. Amyloid Precursor Protein/presenilin-1 (APP/PS1) mice supplemented with GSPE (100 mg/kg) showed a disruption of tau protein hyperphosphorylation and a decrease in Aβ peptide production. These mice also displayed an enhanced capacity for learning, cognition, and memory. These results suggest that GSPE could be a potential protective agent in the treatment of Alzheimer’s disease (AD), as AD is characterized by increased Aβ, hyperphosphorylation and tau protein aggregation [109].

A study was conducted to measure the effects of GSPE containing 85.22% oligomeric proanthocyanidins and 11.16% monomers on a model using streptozocin (STZ) to mimic AD in vitro and in vivo. The findings indicated that administering 50 µg/mL of GSPE as a pre-treatment to STZ-induced primary mouse cortical neurons reduced the level of apoptosis by 55%. In addition, supplementing STZ-treated C57BL/6, Swiss albino, or Kunming mice with 200 mg/kg of GSPE resulted in the repair of oxidative stress, reducing levels of APP, p-tau, and Aβ by 32%, 40%, and 42%, respectively, and achieving a decrease in cognitive impairment. Furthermore, GSPE showed inhibitory potential on STZ-induced glycogen synthase kinase 3β (GSK-3β), which is involved in oxidative stress, achieved by increasing the phosphorylation of this kinase at Ser-9 [110].

A comparative in vitro and in vivo study was conducted to evaluate the potential AD protective effects of proanthocyanidins in relation to their mean degree of polymerization (mDP). A peanut skin proanthocyanidins (PSP) extract with an mDP of 6.74 was depolymerized to obtain an extract (OPSP) with a lower mDP of 2.87. The effects of both extracts (0.2 mg/mL) on scopolamine-treated SH-SY5Y cells were examined and results showed the OPSP extract to exhibit higher inhibition of amyloid-beta (Aβ_42_) fibrillogenicity and Aβ_-42_-induced apoptosis than PSP extract. This latest extract resulted in a 54% reduction in apoptosis, while the OPSP extract was able to completely prevent apoptosis restoring cell viability to the control level. In addition, OPSP extracts (40 mg/kg) was evaluated on a scopolamine-induced AD model in Sprague- Dawley rats. The results showed that this extract exhibited inhibition of IL-1β, TNF-α, and IL-6 inflammatory factors, by 12%, 18% and 25%, respectively. It also reduced Aβ_42_ plaque deposition and improved the rats’ memory performance. The study suggests that a lower mDP favors proanthocyanidins bioavailability, enhancing their ability to exert their bioactive AD protective properties [94].

The presence of type-A procyanidins in the OPSP extract along with the results achieved using GSPE extracts mainly composed of type-B procyanidins, indicates the potential of these various proanthocyanidin structures as neuroprotective agents. The bioactivity findings described at the extract level and as individual compounds, coupled with their low cytotoxic effects, need to be further complemented with studies to elucidate their action mechanisms, fully assess their safety and explore their potential application as neuroprotective agents.

## 5. Anthocyanins

Anthocyanins are commonly used as natural pigments and additives in the cosmetics and food industries, as well as in dietary supplements [88]. The term anthocyanin is derived from the Greek words Anthos, meaning flower, and kyanos, meaning blue [111]. Anthocyanins are a diverse class of water-soluble flavonoid pigments responsible for the red, purple, and blue coloration observed in a wide variety of fruits, vegetables, and flowers. They are abundant in various plant sources, including berries, such as blueberries (*Vaccinium sect. Cyanococcus*), blackcurrants (*Ribes nigrum*), elderberries (*Sambucus nigra*), chokeberries (*Aronia* spp.), and blackberries (*Rubus fruticosus*), as well as grapes, red cabbage (*Brassica oleracea*), purple sweet potatoes (*Ipomea batatas* (L.) Lam), and black rice (*Oryza sativa* L.) [112].

**Figure 4:**
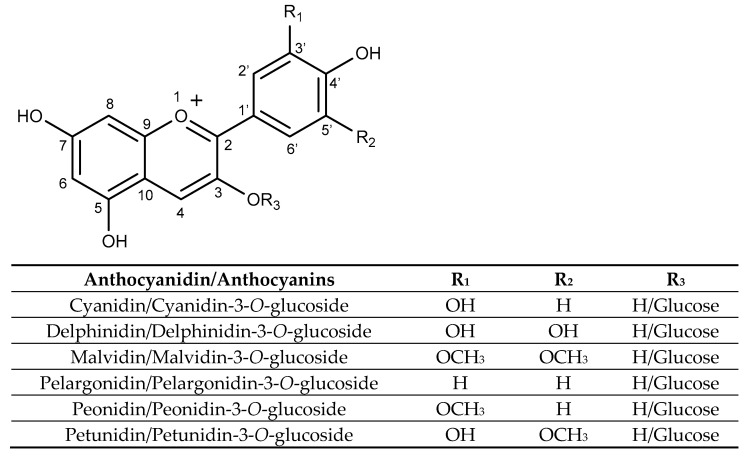
Structure of selected anthocyanidins and their glucosides anthocyanins.

Structurally, anthocyanins are glycosylated forms of anthocyanidins, with a flavylium ion backbone. Their substitution patterns and attached sugar moieties influence their stability, colour intensity, and biological activity. As shown in Figure 4, anthocyanins are characterized by a carbon structure composed of two aromatic rings (A and B rings) and a heterocyclic pyran ring (C ring) fused to the A ring. Glycosylation predominantly occurs at the hydroxyl groups at carbons 3, 5, or 7 [113].

Some important anthocyanins are cyanidin, delphinidin, pelargonidin, malvidin, peonidin, and petunidin, each varying in the number and position of hydroxyl and methoxy groups on the B-ring and the carbohydrate moiety [114]. Anthocyanin composition varies across different sources, for example, malvidin-3-glucoside is mainly found in grapes and red wine, while cyanidin-3-glucoside is abundant in black rice and blackberries [115].

The stability of anthocyanins varies significantly depending on their molecular structure. Compounds like delphinidin and cyanidin, which have reactive hydroxyl groups, typically exhibit greater stability compared to peonidin, malvidin, and petunidin. Additionally, glycosylation at carbon 3 tends to stabilize these molecules, while glycosylation at carbon 5 generally decreases their stability. Acylation further enhances anthocyanin stability, promotes intramolecular pigmentation and facilitates self-association reactions [115].

### 5.1. Antioxidant Activity

The significant antioxidant properties of anthocyanins are attributed to their high electron-donating capability, which allows them to effectively scavenge free radicals such as reactive oxygen (ROS) and nitrogen species. Their structural characteristics enhance their ability to donate hydrogen atoms or electrons to counteract ROS, which is essential for their protective effects against diseases caused by oxidative stress [116,117,118]. The flavylium cation structure is crucial as it enables the delocalization of radical electrons across sp^2^ orbitals in the oxonium group, thereby facilitating the scavenging of free radicals and reducing ROS formation [119].

Important structural factors include phenolic hydroxyl groups, with the number and position of these groups and methoxy groups influencing radical stabilization and scavenging potential. Higher levels of hydroxylation, especially in the *para*- and *ortho*-positions, are generally associated with greater antioxidant efficiency, as they stabilize semiquinone radicals formed during antioxidant activity [120]. Specifically, hydroxyl substituents at positions 3, 5, and 7 (rings A and C) and 3′, and 4′ (ring B) significantly enhance electronic delocalization, leading to increased stability of anthocyanin radicals and enhancing antioxidant effectiveness [121]. Furthermore, the presence of catechol moieties (3′,4′-dihydroxyphenyl groups) on the B-ring significantly boosts antioxidant activity by forming *ortho*-semiquinones and *ortho*-quinones through sequential single-electron transfer reactions [121,122].

Other structural modifications such as glycosylation particularly at position 3, tend to diminish antioxidant effectiveness because it reduces electron delocalization capabilities. In contrast, acylation with phenolic acids considerably enhances antioxidant activity through improved radical stabilization. Diacylation further amplifies these effects, whereas glycosylation at position 5 tends to negatively impact antioxidant efficacy [120].

Among the six most common anthocyanidins, those with higher hydroxy substitution, such as delphinidin with its pyrogallol unit, cyanidin and petunidin with an *ortho*-dihydroxyl substitution, have shown higher antioxidant values compared to those with only one hydroxyl group, such as malvidin, peonidin and pelargonidin [123]. For instance, a study comparing antioxidant activity using the DPPH assay, found that EC_50_ values were 3.75 μM for delphinidin, corresponding to the highest radical scavenging potential, followed by cyanidin with a close value of 3.85 μM. Pelargonidin had a value of 5.25 μM, indicating lower antioxidant potential [124].

Another study using the ORAC assay, indicated that malvidin had a 30.5% and 18.7% higher antioxidant capacity than peonidin and pelargonidin, respectively [125]. This aligns with the expected role of methoxy groups in malvidin and peonidin contributing to radical delocalization stability, while pelargonidin, lacking such functional groups, is expected to have the lowest antioxidant capacity. Additionally, findings from a study using the TEAC assay, showed that delphinidin and petunidin were the best antioxidant compounds with TE values of 3.91 and 3.58 μM, respectively, followed by cyanidin with a TE of 3.47 μM. Meanwhile, malvidin, peonidin and pelargonidin, lacking the catechol structure, showed lower TE values of 2.95, 2.40 and 2.26 μM, respectively [119], suggesting the contribution of hydroxy and methoxy groups to the radical stabilization mechanism.

Comparative results from FRAP assays indicated a similar trend, with petunidin having a higher TE value (2.76 μM) than delphinidin (2.53 μM), while pelargonidin showed lower antioxidant activity with a TE value of 1.09 μM. When using the DPPH assay, delphinidin and petunidin still showed the best TE values of 1.69 and 1.61 μM, respectively, and pelargonidin had the lowest TE value (0.71 μM). However, the results indicated that malvidin had a slightly better TE value (1.33 μM) than cyanidin (1.30 μM) [119].

Overall, these results demonstrate some variability in the expected relationship between anthocyanins’ structure and antioxidant activity. However, delphinidin and petunidin consistently emerge as the most active compounds against radicals, while pelargonidin exhibits the least radical scavenging activity. The structural features and mechanisms of anthocyanins highlight their significant potential as bioactive antioxidants with promising therapeutic implications. Their antioxidant properties contribute to their anti-inflammatory and neuroprotective effects, as demonstrated in numerous studies summarized in the following sections.

### 5.2. Anti-Inflammatory Effects

Anthocyanins exhibit significant anti-inflammatory effects. For example, a blueberry anthocyanin-rich fraction primarily composed of malvidin-3-glucoside (Mv-3-glc) and malvidin-3-galactoside (Mv-3-gal), followed by delphinidin-3-glucoside (Del-3-glc) and Petunidin-3-glucoside (P-3-glc), was found to inhibit TNF-α inflammation-driven adhesion of THP-1 monocytes on human umbilical vein endothelial cells (HUVECs). This fraction showed the highest activity at 10 μg/mL, resulting in 60.2% inhibition, which was lower than adhesion levels without TNF-α stimulation. Individual anthocyanins, such as Mv-3-glc, Del-3-glc and cyanidin-3-glucoside (Cy-3-glc) were also tested. Results showed that Cy-3-glc was the most effective at a concentration of 10 μg/mL, with a 41.8% inhibition, while Mv-3-glc had a 33.9% inhibition, and Del-3-glc did not show a significant effect. This highlights the synergistic role of different anthocyanins in the anti-inflammatory effect of the blueberry anthocyanin-rich fraction [126].

Another study investigates the impact of supplementing the diet of apolipoprotein E-deficient (apoE^−/−^) mice with 1% blueberry anthocyanins powder. The results showed an 86.6% and 98.9% inhibition, respectively, in the expression of LPS-stimulated inflammatory cytokines TNF-α and IL-6 [127]. Other studies have also demonstrated the anti-inflammatory potential of malvidin (Mv) and its glycosides [128]. For instance, Mv, Mv-3-glc and Mv-3-gal were found to be associated with the inhibition of IκBα degradation in HUVECs stimulated with TNF-α. Cells pre-treated with a 10 µM concentration of each anthocyanin or an equimolar mixture of the two Mv glycosides were found to upregulate IκBα expression with the mixture of Mv-3-glc and Mv-3-gal showing the highest (~80%) inhibition value [129,130].

A different study on LPS-stimulated peripheral blood mononuclear cells (PBMCs) regarding IL-6, TNF-α, IL-1β cytokines production, indicated that a pre-treatment with Mv (100 µM) produced an 84.3%, 58.3% and 78.7% inhibition, respectively, and that LPS-upregulated COX-2 expression was reduced by 79.2%. In addition, LPS-downregulated IL-10 anti-inflammatory cytokine improved to normal levels with 25 µM malvidin pre-treatment and showed a 1.8-fold increase with 100 µM Mv [131]. Another study using PBMC CD23-activated monocyte-derived macrophages (MDM), showed that Mv-3-glc (100 µM) isolated from a grape anthocyanin extract was able to inhibit TNF-α and IL-1β cytokines secreted by the CD23-stimulated MDM by 25% and 65% without showing toxicity against PBMC [132].

Meanwhile, a study on LPS-stimulated human monocytic cells THP-1 demonstrated that Mv at a dose of 200 µM effectively decreased the production of pro-inflammatory cytokines, including IL-1β, TNF-α, and IL-6, achieving between 50 and 66% inhibition. Concurrently, it increased the production of the anti-inflammatory cytokine IL-10 in LPS-stimulated THP-1 cells [133]. Another study on bovine arterial endothelial cells (BAEC) showed that Mv-3-glc (25 µM) decreased the 3.5-fold increase in iNOS observed when BAEC cells were pre-treated with peroxy-nitrite by around 40%. Additionally, Mv-3-glc suppressed the 1.7-fold increase in IL-6 production and the 2-fold growth of COX-2 expression induced by the peroxy-nitrite treatment [134].

Malvidin (Mv) has been shown to modulate critical inflammatory signaling pathways in both immune and non-immune cells [135]. For example, in LPS-stimulated RAW264.7 macrophages, Mv (50 µM) reduced inflammatory responses by inhibiting MAPKs, particularly JNK phosphorylation (70%), leading to decreased activation of the NF-κB pro-inflammatory transcription factor [136]. Another study examined the impact of Mv on stress-induced premature senescence in WI-38 cells (human lung-derived diploid fibroblast cells) pre-treated with hydrogen peroxide (H_2_O_2_). The study found that Mv (10 µg/mL) decreased iNOS expression by 65%, COX-2 expression by 78%, and NF-κB production by 58%, indicating protective effects against inflammation induced by oxidative stress [137].

Finally, a study on monosodium iodoacetate-induced osteoarthritis Wistar rats indicated that Mv (20 µg/mL) inhibited pro-inflammatory cytokines IL-6, IL-1β and TNF-α by 38%, 47% and 70%, respectively. It also suppressed the p65-induced transcription of IL-6 without affecting IL-6 transcription, suggesting an IκBα-independent downregulation of the NF-κB signaling pathway [138]. These findings reinforce the broad anti-inflammatory effects and the potential of anthocyanins as therapeutic agents in managing inflammatory conditions.

### 5.3. Neuroprotective Potential

Studies have shown that anthocyanins have protecting effects against neuronal injuries, like ischemia, and neurodegenerative diseases, such as Parkinson’s disease, and Alzheimer’s disease [139]. For example, the effects of a purified mixture of anthocyanins from the seed coat of black soybeans, which included cyanidin-3-glucoside (68.3%), delphinidin-3-glucoside (25.2%), and petunidin-3-glucoside (6.5%) were studied on H_2_O_2_-treated SK-N-SH human neuroblastoma cells. The findings indicate that pre-treatment of these cells with 25 µg/mL of the anthocyanin mixture reduced apoptosis by up to 92.9%, decreased intracellular ROS levels by 51.3% and reduced the phosphorylation of Apoptosis signal-regulating kinase 1 (ASK1), JNK and p38 MAPK by 3.0-fold, 2.7-fold and 1.4-fold, respectively, thereby inhibiting the activation of the ASK1–JNK/p38 pathways [140].

A study on an ethanol-exposed C57BL/6 mice model showed neuroprotective effects exerted by Cy-3-glc treatment. Administering Cy-3-glc purified from blackberries at a dose of 10 mg/kg significantly decreased ethanol-induced activation of caspase-3 and reduced the number of Fluoro-Jade C-positive cells, indicating a decrease in apoptosis and neurodegeneration caused by ethanol exposure. Additionally, Cy-3-glc inhibited ethanol-mediated activation of glycogen synthase kinase 3β (GSK3β), which is known to cause neuronal death, by promoting ser9 phosphorylation and reducing tyr216 phosphorylation. Furthermore, Cy-3-glc treatment suppressed ethanol-induced expression of p47phox, a key regulatory protein of NADPH oxidase, and MDA [141]. These findings suggest the potential of Cy-3-glc in combating ethanol-related neurodegeneration, which could lead to central nervous system (CNS) malformations and mental retardation. However, further research is necessary to fully understand the efficacy and safety of Cy-3-glc in this health condition.

Another study examined the neuroprotective properties of cyanidin (Cy) and Cy-3-glc, against oxidative stress induced by H_2_O_2_ in human neuroblastoma SH-SY5Y cells. Treatment with 100 μM concentrations of both compounds resulted in a significant decrease in H_2_O_2_-induced ROS formation, with Cy showing more inhibition (75%) than Cy-3-glc (50%). While Cy does not exhibit antioxidant effects at the cytosolic level, it did enhance the total antioxidant capacity in intracellular and extracellular fractions of the cells and inhibited hydrogen peroxide-induced DNA fragmentation [142]. These findings suggest that the neuroprotective effects of Cy and Cy-3-glc may depend on their bioavailability, their interaction with the cellular membrane, and their uptake into neuronal cells. These structural characteristics appear to be crucial for the preservation of mitochondrial integrity, as well as the level of antioxidant defenses and neuroprotection.

Several studies have demonstrated the cognitive benefits of anthocyanins, particularly in enhancing memory and learning. For instance, the effects of malvidin-3-glucoside (Mv-3-glc) were studied in LPS-treated murine cortical cells in relation to the response of nucleotide-binding domain leucine-rich repeat-containing proteins (NLRs) and other inflammasomes. Treatment with Mv-3-glc (10 μM) showed inhibition of NLRP3, NLRC4 and melanoma 2-like receptor (AIM2), resulting in a reduction of NLRs and AIM2-mediated secretion of the IL-1β inflammatory cytokine. Additionally, treatment of C57BL/6J male mice with Mv-3-glc (12.5 mg/kg) was shown to mitigate anxiety and depression-like behaviour in a mouse model of chronic unpredictable stress (CUS) [143]. These findings support the role of Mv-3-glc in modulating inflammasome activation in vitro and in mitigating CUS-induced behavioural impairments in vivo, thus providing beneficial effects to counteract anxiety and depression.

Another in vivo study supporting these findings involved healthy *Balb-c* mice treated with an anthocyanin-rich blueberry extract (60 mg/kg). This treatment led to a significant increase in brain glutathione (GSH) and ascorbate levels, decreased AChE activity, and improvements in learning and motivational behaviours [144]. Additionally, administering Cy-3-glc (30 mg/kg) to APPswe/PS1ΔE9 transgenic mice downregulated TNF-α, IL-1β, and IL-6 pro-inflammatory cytokines by 45–55%, reduced the levels of Aβ (Aβ_40_ and Aβ_42_) plaques, and enhanced learning and memory. These behavioural improvements suggest the efficacy of anthocyanins in neurodegenerative conditions [145].

Anthocyanins have demonstrated protective effects against cerebral ischemia by attenuating oxidative damage and preserving neuronal function. For instance, a study conducted on oxygen-glucose deprivation (OGD) induced in Sprague Dawley rat primary cortical neurons revealed a 13.9% increase in ROS-positive cell counts. Treatment with a purified extract of black soybean seed coat anthocyanins (10 μg/mL), containing Del-3-glc, P-3-glc and Cy-3-glc, reduced ROS production to 1.9%. Furthermore, OGD exposure led to a decrease in mitochondrial membrane potential (MMP) fluorescence intensity to 28.4%, while the anthocyanin extract (10 μg/mL) was able to maintain 63.7% in those OGD-exposed primary cortical neurons. These findings suggest protective effects on neurons against ischemic damage [146].

In addition, a study focused on the effects of Cy-3-glc purified from tart cherries (*Prunus cerasus*) on C57BL/6 mice undergoing permanent middle cerebral artery occlusion (pMCAO). The results indicated that pre-treatment with Cy-3-glc (2 mg/kg) significantly reduced brain superoxide levels and decreased infarct volume by 27%. Further, Cy-3-glc delayed treatment after pMCAO followed by two supplementary doses (2 mg/kg) also led to a 25% reduction in infarct volume [147]. These findings support the potential of these compounds in mitigating ischemic brain injury by modulating oxidative stress pathways and highlight their antioxidant-mediated neuroprotective mechanisms.

Parkinson’s Disease (PD), as mentioned earlier, is a common neurodegenerative disease in the CNS with no effective therapeutic strategies to date [148]. Anthocyanin-rich botanical extracts have shown neuroprotective effects in models of Parkinson’s disease. For example, a dose of 30 ng/mL of blackcurrant extract rich in anthocyanins (94.4%), primarily containing cyanidin 3-*O*-rutinoside, delphinidin-3-*O*-rutinoside, Del-3-glc, and Cy-3-glc, as well as individual anthocyanins Mv-3-glc, Del-3-glc and Cyanidin-3-*O*-sophoroside (1 μM) displayed a significant protective role against rotenone-induced dopaminergic neuronal death, a model for Parkinsonian neurodegeneration, improving the dopaminergic cell survival by 70% [149].

In addition, a clinical study showed that supplementation with blackcurrant extract was associated with increased concentrations of cyclic glycine-proline (cGP), a neuropeptide linked to neuroprotection and cognitive function, in the cerebrospinal fluid (CSF). In a clinical intervention, blackcurrant concentrate capsules containing 35% anthocyanins were administered at a dose of 300 mg twice daily over a four-week period, resulting in a 65% increase in CSF cGP levels [150]. These findings suggest that dietary anthocyanins may offer therapeutic benefit in modulating neurodegenerative processes associated with Parkinson’s disease.

A growing body of research highlights the therapeutic potential of anthocyanins in mitigating the pathophysiological features of Alzheimer’s disease (AD), the most common cause of dementia, affecting approximately 50 million people worldwide [151]. For instance, anthocyanin extracts from Korean black beans have been shown to enhance the GSK3/Akt/Phosphoinositide 3-kinase (PI3K) signaling pathway and activate the Nrf2/HO-1 antioxidant defense system, providing neuroprotection in Alzheimer’s models. In fact, a 30-day administration of anthocyanins derived from Korean black beans—consisting of Cy-3-glc (67%), Del-3-glc (25%), and P-3-glc (5%)— to transgenic APP/PS1 mice, led to the upregulation of p-PI3K, p-Akt and p-GSK3β (Ser9) expression, an increase in Nrf2/HO-1 reactivity, and a significant improvement in memory performance [152].

Similarly, in vitro studies involving a formulation composed of equimolar amounts of Cy-3-glc, Mv-3-glc, pelargonidin-3-*O*-glucoside and peonidin demonstrated protective effects against acrolein-induced damage in SK-N-SH neuroblastoma cells. Acrolein induced over 80% cell death. However, treatment with the formulation (20 μM) showed a three-fold inhibition of acrolein-induced SK-N-SH cells death and prevented GSH depletion [153]. Furthermore, a clinical study consisting of a 12-week controlled trial intervention with anthocyanin-rich cherry juice (200 mL/day) improved memory and cognitive function, including verbal fluency and both short- and long-term memory, in older adults with mild-to-moderate dementia [154].

In addition, another study demonstrated that Mv and Mv-3-glc effectively reduced amyloid-β-mediated neurotoxicity induced in mouse neuroblastoma neuro-2A cells by a combination of Aβ_25–35_ and A_β1–40_. Both anthocyanins, at a concentration of 50 μM, improved the downregulation of apolipoprotein E (ApoE) and β-secretase and inhibited the Aβ-induced ROS formation. Mv exhibited a higher inhibition rate (43%) compared to Mv-glc (35%) [155]. Further studies demonstrated that Cy-3-glc can also decrease amyloid-β-induced neurotoxicity in SH-SY5Y human neuroblastoma cells. For example, pre-treatment of SH-SY5Y cells with 100 μM Cy-3-glc reduced apoptosis and necrosis caused by Aβ_25–35_ oligomers aggregation by 38% and 44%, respectively. Similar results were observed with co-treatment using the same dose of Cy-3-glc. This study suggested that the specific structure of anthocyanins and the hydroxyl groups forming hydrogen bonds with the acceptor and donor groups of amino acid side chains present in Aβ_25–35_ peptides, such as methionine, may contribute to the inhibition of aggregation and neurotoxicity [156]

Overall, these results underscore the therapeutic potential of anthocyanins in promoting neuroprotection. The findings suggest that both aglycone and glycosylated anthocyanins confer neuroprotective benefits in cognitive health, ischemia and neurodegenerative diseases such as Parkinson’s and Alzheimer’s. These benefits are achieved through mechanisms involving antioxidant and anti-inflammatory regulation, as well as the inhibition of β-amyloid neurotoxicity, ultimately leading to cognitive enhancement.

## 6. Scope and Limitations

Notwithstanding the strong evidence of in vitro neuroprotection of flavonoids, their low bioavailability is a factor affecting their in vivo effects [157]. In addition, clinical studies on the efficacy and safety of flavonoids are scarce despite being crucial for an accurate evaluation of their potential as therapeutic agents [158]. Therefore, this section addresses efforts to overcome these limitations, including studies on flavonoids interactions with the body microbiota to understand their mechanisms of action. This understanding is crucial for designing safe clinical studies as well as the use of special delivery systems aimed at improving their bioavailability.

Among clinical studies involving flavonols, a randomized clinical trial (RCT) with a double-blind, placebo-controlled design (*n* = 201), showed that a daily treatment with 320 mg of anthocyanins from bilberry and black currant resulted in statistically significant improvement in cognitive function for individuals with high levels of inflammatory biomarkers compared to placebo at 24 weeks. However, there was no significant improvement in the group with low inflammatory biomarkers [159]. This indicates the anti-inflammatory mechanism of anthocyanins extracts when exerting neuroprotection.

A RCT with a double-blind, crossover, placebo-controlled design (*n* = 92) evaluated the impact of daily intake of an encapsulated concentrate of vegetable, fruit, and berry juice powders, rich in the four types of polyphenols reviewed in the present study. The results indicated significant improvement in cognitive performance using the Stroop Test and Reynolds Intellectual Screening Test, compared to the placebo. Additionally, the plasma levels of brain-derived neurotrophic factor (BDNF) and cAMP response element-binding protein (CREB) markedly increased [160], indicating neuroprotective effects of polyphenol intake from food concentrates.

Another RCT on older adults with no cognitive issues (*n* = 90) evaluated the effects of cocoa drinks containing 993, 520, or 48 mg of flavan-3-ols administered during an 8-week period, using the Mini-Mental State Examination (MMSE), the Trail Making Test (TMT) A and B, and the Verbal Fluency Test (VFT). The response of MMSE scores to the different treatments did not differ but there was an impact on specific aspects of cognitive function improvement, with TMT scores significant higher (*p* < 0.0001) for the first two flavanol doses compared to the low dose. There was also a significant difference (*p* < 0.0001) in the VFT values among all treatment groups [161,162]. Additionally, a double-blind RCT (*n* = 26) administering 30 mL/day of blueberry concentrate containing 387 mg of anthocyanidins for 12 weeks found improvement (*p* < 0.05) in working memory (2-back test) between blueberry treatment groups and placebo groups [163,164].

A double-blind, placebo-controlled, crossover RCT study was conducted with 36 healthy participants aged 18–35. They were supplemented with either blackcurrant extract at a dose of 8.05 mg of anthocyanins/kg or blackcurrant juice at a dose of 7.79 mg of anthocyanins/kg. This was done in a repeated measures design with three sessions at least seven days apart. During each session, participants completed seven repetitions of the digit vigilance task, Stroop task and rapid visual information task. The study found that both the anthocyanins extract and juice improved reaction time, sustained attention and psychomotor speed [165].

Similarly, another RCT with a double-blind, placebo-controlled crossover design assessed the effects of acute administration of anthocyanin-rich blackcurrant juice standardized at 500 mg of polyphenols. This was consumed twice with at least one week wash-out period in between. Results from cognitive tasks using the CogTrack™ (Wesnes Cognition Limited, Northampton, NN1 5AN, UK) showed improved alertness, reduced fatigue, and enhanced reaction times. This suggests the extract positively impact mood, attention, and overall cognitive outcomes [166].

On an individual compound level, a prospective, single-arm, open-label phase 1/11 clinical trial evaluated the efficacy and safety of epigallocatechin gallate (EGCG) aerosol in controlling COVID-19 pneumonia in cancer patients (*n* = 60). The study found that EGCG aerosol let to a 56.4% improvement rate in pneumonia and an 82% incidence of non-progression of pneumonia. Safety assessments based on the Common Terminology Criteria for Adverse Events (CTCAE) v. 5.0, showed that at a dose of 8817 µmol/L of ECGC, two patients experienced grade 1 adverse events of stomach discomfort and nausea, which resolved within an hour [167]. This suggests that EGCG aerosol treatment is well-tolerated and can improve pneumonia conditions in patients.

In addition to the scarcity of clinical trials, the lack of standardization is a limitation for their comparability. For example, there is a wide variability in daily dosages of flavonoids, with trials using EGCG reporting ranges from 200 to 2000 mg/day. There is also disparity in the measurement methodologies, with some trials using biomarker quantification while others use cognitive scales. For instance, it was reported that only one third of flavonoid trials for Alzheimer’s disease involved standardized biomarkers, such as p-Tau protein or cerebrospinal fluid Aβ_42_ [168].

Notably, the safety of flavonoids for therapeutic purposes is an underestimated field of research. Flavonoids from food sources are classified as low-risk compounds in animals, with LD_50_ between 2 and 10 g/kg in rats for most flavonoids [169]. However, concerns regarding their potential toxicity include interactions with drug-metabolizing enzymes, and the lack of clinical studies to establish the safety of flavonoid intake in foods and as dietary supplements [170]. For example, a cross-sectional study (*n* = 1183) using a validated 215-item food frequency questionnaire quantified flavonoid intake. The results showed a total absolute flavonoid intake of 626 mg/day, with flavan-3-ols, mainly from tea, contributing to 86.5% of total flavonoids consumed, followed by anthocyanidins (5.3%) from berries. Flavonols, mostly from wine and fruits, contributed 4.8%, and flavones primarily from vegetables accounted for 0.2% of total consumed flavonoids. However, further investigation is needed to assess actual health benefits and safety [171].

As mentioned, an important safety factor to consider is the potential interactions of flavonoids that can affect other metabolites. For example, EGCG has been reported to reduce the bioavailability of folic acid due to its interaction with the folate transporter. This is a concern for pregnant women and individuals for whom a reduction in folic acid levels may have clinical consequences. They are advised against consuming green tea [172,173]. EGCG has also been shown to inhibit the intestinal absorption of non-heme iron in a dose-dependent manner in a controlled clinical trial [174]. This can impact patients with poor iron status. Furthermore, despite EGCG’s synergistic effects in enhancing the properties of anticancer drugs, there is evidence that green tea under specific conditions related to the patient’s metabolism, can induce oxidative stress in the liver, leading to hepatotoxicity [175].

Interactions of flavonoids with P-glycoprotein (P-gp) and drug-metabolizing enzymes like Cytochrome P450 3A4 (*CYP3A4*) are important factors to consider. For example, quercetin has been found to inhibit P-gp, potentially causing drug–drug interactions by affecting drug pharmacokinetics [176]. A study on the effect of oral quercetin on oral and intravenous doxorubicin in male Sprague-Dawley rats revealed that quercetin inhibited cytochrome P450 3A4 (*CYP3A4*) enzyme activity in a concentration-dependent manner with an IC_50_ of 1.97 µM. This led to an increase in the peak plasma concentration of oral doxorubicin, resulting in higher absolute bioavailability compared to the control group. The relative bioavailability of oral doxorubicin was also increased by 1.32 to 2.36-fold. However, intravenous doxorubicin was not affected by quercetin. Therefore, concurrent use of quercetin can improve the bioavailability of oral doxorubicin [177].

Interactions of flavonoids have been studied in an effort to overcome pharmacoresistance to CNS-related drugs such as antiepileptics (AED) caused by the overexpression of P-gp at the blood–brain barrier (BBB). An in vitro strategy was implemented to combine flavonoids with AEDs like carbamazepine, oxcarbazepine, and phenytoin using Madin-Darby canine kidney II (MDCK II) cells, as well as those transfected with the human multidrug resistance-1 (MDR1) gene, which overexpresses the P-gp (MDCK-MDR1). The findings indicated that kaempferol, (−)-epigallocatechin gallate, and quercetin, along with other flavonoids, had the most significant impact on increasing the intracellular accumulation of rhodamine 123 in MDCK-MDR1 cells. This effect potentially occurred through the inhibition of P-gp activity and led to a notable increase in the intracellular accumulation of these AEDs [178].

Related to the neuroprotective potential of flavonoids in brain disorders, their capability to prevent thrombosis has been demonstrated. For instance, it has been shown that flavan-3-ols from red wine and grape extracts enhance interactions with the platelet-endothelial cell adhesion molecule-1 (PECAM-1/CD31), which possesses anticoagulant properties [179,180]. A study on quercetin interaction with warfarin indicated that the flavonoid could displace the drug due to its higher affinity for binding to human serum albumin, potentially leading to interference with warfarin therapy at high quercetin doses [181]. In summary, while the simultaneous administration of flavonoids and conventional drugs may offer potential benefits, it also raises concerns about potential toxicity at high doses and excessive consumption as dietary supplements. By impacting the plasma concentrations of drugs, flavonoids can either result in a drug overdose or a diminished therapeutic effect, underscoring the necessity for further comprehensive studies [170].

Regarding the bioavailability of flavonoids, it is important to consider their bioaccessibility, which involves their release from the food matrix and transformation into an absorbable form before entering the bloodstream [182]. When flavonoids are consumed in food rather than as concentrated extracts, the interaction of flavonoids with other food components becomes a significant factor. Research has shown that digestible carbohydrates, lipids, vitamins and other flavonoids can increase flavonoid bioavailability by activating transporters and enhancing micellization. Conversely, dietary fiber, proteins, and minerals have been found to decrease flavonoid bioavailability by potentially causing increased retention of flavonoids in the food matrix [183,184].

Once in an absorbable form, flavonoid glycosides can undergo phase I transformations, such as hydrolysis in the intestine by enzymes like human cytosolic β-glucosidase (CBG) or lactase phlorizin hydrolase (LPH) and phase II conjugations in epithelial and liver cells by enzymes like catechol-*O*-methyltransferases (COMT), uridine-5′-diphosphate-glucuronosyl transferases (UGT), or sulfotransferases to produce methyl, glucuronide and sulfate forms, respectively. These processes along with flavonoids’ interactions with the gut microbiota enable their metabolites to circulate and exert their biological activities [185,186]. The high variability in gut microbial metabolites and the particular structure of flavonoids impact their conjugation and degradation pathways, therefore affecting their efficacy and safety.

Among the flavonoid groups discussed in this review, anthocyanins have been reported to be the most rapidly absorbed with poor efficiency and fast elimination. After daily administration of 150 mg to 2 g anthocyanins, concentrations in plasma were found to be in a range from 10 to 50 nmol/L, with excretion ranging from 0.004% to 0.1% of the intake after 1.5 h and reaching Cmax at an average of 2.5 h. In contrast, flavonol glycosides like those of quercetin have been reported to have longer half-lives ranging from 11 to 28 h, potentially leading to increased accumulation in plasma through repeated intakes. For example, plasma concentration increased from 50–80 nmol/L overnight to 0.63 and 1.5 µmol/L after daily supplementation of quercetin with 80 mg and 1 g for 7 and 28 days, respectively. Among flavan-3-ol monomers, ECGC has shown the highest presence in plasma in free form, ranging from 77 to 90%, while (epi)catechin is present as glucuronides or sulfate conjugates. Finally, proanthocyanidin oligomers due to their polymerization, exhibit almost 100 times less absorption than monomers. However, their health effects have been attributed to their direct interaction with the microbiota [8,157].

Another factor the genetic polymorphisms exhibited by the metabolic enzymes mentioned above, which can be influenced by diet and impact the metabolism and distribution pharmacokinetics of flavonoids. For example, most quercetin *O*-glycosides are hydrolyzed by LPH but are not substrates for CBG. Their methylated metabolites are influenced by COMT genetic polymorphism as well as by the cytochrome P450 demethylation specificity at the 4′ position. Additionally, the levels of UGT, which catalyze the transformation to glucuronic acids, are affected by diet, genetic polymorphism and environment. This accounts for the differences observed among individuals in the glucuronidation of flavan-3-ol monomers like catechin. Furthermore, flavones like luteolin-7-*O*-glucoside are first deglycosylated before their conversion to glucuronides. However, it has been reported that after consuming red berries, anthocyanins glycosides, such as cyanidin-3-glucoside and cyanidin-3,5-diglucoside were found in human plasma [187].

The key role of gut microbiota variability is supported by numerous studies. For instance, the C-glycosylated flavone vitexin transformation relies on specific bacteria in the colon for the crucial deglycosylation step, which is carried out by the intestinal Lachnospiraceae bacteria, while resistant to other strains. Ring fission and dehydroxylation reactions are then achieved for further degradation into small phenolic acids [188]. Quercetin glycosides are broken down into cyclic fission products such as 3,4-dihydroxyphenylacetic acid (DOPAC), which is later dehydroxylated into 3-hydroxyphenylacetic acid and protocatechuic acid (PCA) [186]. Additionally, proanthocyanidins are metabolized by intestinal bacteria to produce 3,4-dihydroxyphenylpentanoic acid, which then breaks down into active metabolites, like 3,4-dihydrocaffeic acid and PCA [189]. Similarly, anthocyanins are converted into p-coumaric acid, vanillic acid and PCA, which possess antioxidant, anti-inflammatory and neuroprotective properties.

Flavonoids can modulate gut microbiota in a two-way interaction pathway. For instance, quercetin has been shown to balance intestinal microbiota in high-fat diet (HFD)-induced non-alcoholic fatty liver disease (NAFLD) mice by reducing the ratio of Firmicutes/Bacteroidetes in the HFD group [190]. Among flavan-3-ols, (epi)gallocatechin gallates from tea extracts have been found to increase *Bacteroidetes* and decrease *Firmicutes* in C57BL/6J mice fed a HFD/high-sucrose diet [191]. In a different study, the administration of blueberry extract to Sprague Dawley rats (4 mL/kg BW/day) for 6 days significantly increased *Lactic acid bacteria* and *Bifidobacteria* [192]. Lastly, a RCT with a crossover, controlled intervention design (*n* = 10) demonstrated that consuming red wine (272 mL/d) for 20 days increased the number of beneficial bacteria and reduced the number of potential pathogenic bacteria in the intestinal flora, showing prebiotic-like effects [193].

As stated earlier, the ability of flavonoids’ metabolites to cross the blood–brain barrier (BBB) is a crucial factor in neuroprotection. Limited studies, mostly using mice, rats and human brain endothelial cell models, indicate that PCA and other small phenolic acids can adequately penetrate the BBB. For example, 5-(3′, 5′-dihydroxyphenyl)-*γ*-amyl lactone, *O*-toluenol, its glucosinolates and sulfate adducts are microbial metabolites from flavan-3-ol monomers like epigallocatechin, capable of crossing the blood–brain barrier and exerting in vitro effects on neuronal proliferation [182]. Another study demonstrated that anthocyanins, flavan-3-ols, flavonols, and their metabolites penetrated a BBB model of hCMEC/D3 cells in a time-dependent manner [194]. However, the low bioavailability of flavonoids, BBB efflux transporters like P-glycoprotein for which flavonoids and their metabolites can act as substrates—as in the case of quercetin—and enzymes like β-glucuronidases that can induce deconjugation, limit their accumulation across the BBB at pharmacologically effective concentrations [195].

To overcome these limitations, special delivery systems and organic multicomponent strategies have been developed with the aim of improving flavonoids’ bioavailability [196]. For example, liposomal formulations prepared using a thin-film hydration method have been shown to increase the circulation of a multicomponent treatment containing the chemotherapeutic drug vicrestin and the flavonol quercetin in a 2:1 molar ratio, enhancing tumor growth inhibition against JIMT-1 human breast cancer cells [197]. In was also reported that loading quercetin into 20 nm lipid nanocarriers made-up of 12-hydroxystearic acid-polyethylene glycol copolymer, lecithin and castor oil resulted in a delayed in vivo release under GTI conditions [198]. Additionally, quercetin-loaded liposomes composed of cholesterol and lecithin, modified with galactosylated chitosan, achieved sustained quercetin release and reduced lipid oxidation in an acute liver injury mice model, thereby protecting the liver from damage [199].

Another liposomal formulation composed of cholesterol, L-α-phosphatidylcholine, and DSPE-PEG was used to co-encapsulate the flavonoids quercetin and EGCG along with doxorubicin. The results indicated improved liposome stability through PEGylation, which extended the drugs’ release time in vitro with significant amounts of drugs released up to 10 days. This formulation exhibited antimicrobial activity against *E. coli* and cytotoxicity in leukemic K562 cell lines [200]. A different strategy involved loading quercetin and rutin into a polymer matrix containing a water-soluble polymer with low molecular weight to enhance water wettability. The results showed that polyethylene glycol and cellulose acetate matrices improved the flavonoids cytotoxicity against HeLa cervix cancer cell lines and achieved higher selectivity compared to normal cells [201].

Bioavailability improvement, aiming at enhancing neuroprotective activity, was assessed by encapsulating EGCG in liposomes made from phosphatidylserine (PS) or phosphatidylcholine (PC), with or without vitamin E (VE), using a hydration and membrane extrusion method. The results showed that EGCG PS-VE-loaded liposomes exhibited the most significant reduction in LPS-induced pro-inflammatory cytokines and restored motor function in a rat model of Parkinson’s disease [202]. A different approach was taken to evaluate the neuroprotective effects of anthocyanins extracted from black soybeans and anthocyanin-loaded poly (ethylene glycol)-gold nanoparticles (PEG-AuNPs) in mouse models of Alzheimer’s disease (AD) injected with Aβ1_42_. The results indicated that anthocyanins loaded with PEG-AuNPs were more effective than free anthocyanins in reducing Aβ1_42_-induced neuroinflammatory and neuroapoptotic markers by inhibiting the p-JNK/NF-κB/p-GSK3β pathway [203,204].

Clinical studies conducted with these systems are even more scarce. A double-blind, randomized, three arm pilot study (*n* = 24) evaluated a multi-organic-material (MOM) system using a single dose of 2.5 g of resveratrol alone or with piperine in doses of 5 mg or 25 mg, and followed-up on adverse events for 30 days. The results indicated only slightly higher Cmax, resveratrol and resveratrol glucuronide concentrations in plasma with piperine. However, the doses did not sufficiently alter the pharmacokinetics to achieve the significant two-fold or higher enhancement observed in a previous murine model [205].

An uncontrolled open case series study on children with autism spectrum disorders (*n* = 37), evaluated a dietary supplement composed of luteolin, quercetin, and rutin in a ratio of 5:3.5:1.5. This supplement was in a liposomal formulation of olive kernel oil, aiming to increase the absorption of flavonoids. After four months, symptoms such as eye contact, social interaction, and resumption of speech improved in 50%, 25% and 10% of children, respectively, with no reported adverse effects [206]. Despite some promising results reported with these delivery strategies, there are challenges to overcome, such as particle stability and scalability for large-scale production, in order to achieve improved bioavailability and enable the potential application of flavonoids in neuroprotection.

## 7. Conclusions

This review contributes to the understanding of the bioactive effects of four important classes of flavonoids that are commonly used as ingredients in dietary supplements. The review focuses on their antioxidant, anti-inflammatory and neuroprotective effects, addressing key health priorities for consumers. In terms of structure–activity relationships in the four types of flavonoids, common characteristics for higher bioactivity include the presence of pyrogallol B-rings. For example, flavan-3-ols like epigallocatechin exhibit higher bioactivity compared to catechin, which has a catechol B-ring. Similarly, in flavonols, myricetin has been found to have higher bioactivity compared to quercetin, while kaempferol, with just one hydroxyl group, shows the lowest effect. Among flavones, luteolin, with a catechol B-ring, also exhibits higher activity than apigenin, which only has an OH in the 4′ position. Anthocyanins also follow this trend, with pelargonidin exhibiting the least activity and delphinidin, with a pyrogallol B-ring, showing higher effects. *O*-glycosylation has been shown to lower the activity of aglycones, while C-glycosylation increases their bioactivity. A higher mean degree of polymerization (mDP) in proanthocyanidins has been shown to inhibit their bioactivity due to their lower absorption.

Among flavonols, isorhamnetin aglycone and rutin glycoside have consistently shown useful neuroprotective effects, primarily due to their anti-inflammatory properties. However, isorhamnetin has lower market penetration compared to rutin and quercetin, which are often used as benchmark antioxidants. In the case of flavones, the presence of C-glycosylation has been found to enhance bioactivities of (iso)vitexin and (iso)orientin, particularly in their cognitive improvement and anti-Alzheimer’s potential effects. Regarding proanthocyanidins, epigallocatechin gallate has shown the most promising antioxidant results, attributed to the hydroxy groups that enhance its radical scavenging activity. Lower degrees of polymerization in oligomers have been linked to greater anti-inflammatory and neuroprotective effects compared to higher polymerization levels, due to increased bioavailability. Among anthocyanins, malvidin and its 3-*O*-glycoside have been found to downregulate inflammatory cytokines and modulate critical signaling pathways. Cyanidin-3-glucoside consistently demonstrates superior neuroprotective activity, reducing oxidative stress, protecting mitochondrial function, and decreasing neuronal apoptosis and DNA fragmentation.

It is evident that there is a significant amount of evidence regarding the in vitro antioxidant and anti-inflammatory activity associated with the potential neuroprotective effects of the four groups of flavonoids discussed in this review. However, despite these promising scientific findings, limitations such as low bioavailability and a lack of clinical studies on their efficacy and safety need to be addressed. The increasing trend in consumption, both as food and dietary supplements, appears to be driven by a growing market emphasis on health prevention concerns, which is a top priority for consumers. This trend underscores the importance of conducting comprehensive studies and further research to fully understand the structure–activity relationship of these compounds, their interactions with the gut microbiota, mechanistic issues, drug interactions, and potential toxicity. Bioavailability concerns are being addressed through special delivery systems, such as nanoparticles to enhance their solubility. However, limitations like the development of scaling-up processes need to be addressed. Additionally, it is essential to gather clinical data that can accurately assess the efficacy and safety of these widely consumed flavonoids in order to confirm the potential application of these promising natural ingredients as therapeutic agents.

## Data Availability

Not applicable.
